# Targeting the HLA-E–NKG2A axis in combination with MS-275 enhances NK cell-based immunotherapy against DMG

**DOI:** 10.1186/s13046-025-03390-y

**Published:** 2025-04-29

**Authors:** Yuxuan Deng, Jinqiu Liu, Zhuonan Pu, Yi Wang, Tian Li, Zhuang Jiang, Luyang Xie, Xiaoli Zhang, YingDan Chen, Mingxu Yang, Chao Du, Shuyu Hao, Nan Ji, Zhengping Zhuang, Jie Feng, Liwei Zhang

**Affiliations:** 1 Department of Neurosurgery, Fengtai District, Beijing Tiantan Hospital, Capital Medical University, Beijing, China; 2 Beijing Neurosurgical Institute, Capital Medical University, Beijing, China; 3 Neuro-Oncology Branch, Center for Cancer Research, National Cancer Institute, National Institutes of Health, Bethesda, MD USA

**Keywords:** Diffuse midline glioma (DMG), Histone deacetylase (HDAC), Natural killer (NK) cell, HLA-E

## Abstract

**Background:**

Diffuse midline glioma (DMG) is an aggressive pediatric brain tumor with limited treatment options. Although natural killer (NK) cell-based immunotherapy is promising, its efficacy remains limited, necessitating strategies to enhance NK cell cytotoxicity. Histone deacetylase (HDAC) inhibition demonstrate potential to enhance NK-mediated killing. However, the combination of HDAC inhibitors and NK cell therapy for DMG remains unexplored.

**Methods:**

Patient-derived DMG cell lines and orthotopic mouse models were used to evaluate the effects of the class I HDAC inhibitor MS-275 on cytotoxicity. NK cell-mediated lysis was measured using both luciferase and calcein AM-based assays. The downstream signaling pathways affected by MS-275 were investigated via RNA-seq, CUT&Tag assay, RT‒qPCR, and chromatin immunoprecipitation with qPCR.

**Results:**

Based on bioinformatic analysis, class I HDACs are identified as therapeutic targets in DMG. The corresponding HDAC inhibitor, MS-275 upregulated NK cell-mediated cytotoxicity pathway through GSEA analysis. Pretreating DMG cells with MS-275 elevated NK cell ligand gene expression and enhanced NK cell-induced lysis. In addition to NK-activating ligands, MS-275 elevated the NK-inhibitory ligand HLA-E, thereby enhancing the efficacy of immunotherapies targeting the NKG2A–HLA-E axis. Mechanistically, MS-275 increased HLA-E expression by promoting STAT3 acetylation at lysine 685. Combining MS-275 with NK cell therapy and blockade of the NKG2A–HLA-E axis extended overall survival in orthotopic mouse models.

**Conclusions:**

This study is the first to demonstrate that HDAC inhibition enhances NK cell-mediated cytotoxicity in DMG. Combining HDAC inhibition with NK cell therapy represents a promising therapeutic strategy for treating DMG by targeting NKG2A–HLA-E axis.

**Supplementary Information:**

The online version contains supplementary material available at 10.1186/s13046-025-03390-y.

## Background

Diffuse midline glioma (DMG) is a fatal high-grade glioma (HGG) that arises in the brainstem. DMGs occur mostly in children and less frequently in adults [1]. Surgical resection of DMGs is impossible because of their critical neuroanatomical location. Radiotherapy can relieve neurological symptoms but does not increase life expectancy [2]. Despite decades of research, little progress has been made in the treatment of DMG. With a median survival of less than one year, DMG is the leading cause of death in pediatric patients with brain tumors [3].

Owing to the potent antitumor effects of NK cells, NK cell immunotherapy has garnered increasing interest, and several clinical trials have been conducted for various tumors [4]. Compared with T cells, NK cells are more effective at killing DMG and represent a promising approach for treating DMG [5]. Natural killer (NK) cells are cytotoxic innate-like lymphocytes with inherent features that enable the recognition and elimination of stressed cells, including tumor cells and infected cells [6]. NK cells are regulated by a dynamic balance between signals transduced by a diverse set of activating and inhibitory receptors bound to their corresponding tumor cell ligands [7]. The major ligands recognized by these activating receptors include UL16-binding protein 1-6 (ULBP1-6), major histocompatibility complex class I chain-related gene A/B (MICA/B), intercellular adhesion molecule 1 (ICAM-1), lymphocyte function-associated antigen 3 (CD58) and the B7 family member B7-H6 [8]. The major inhibitory ligands include human leukocyte antigen E (HLA-E, which binds to NKG2A) and the B7 family member B7-H3 (CD276). Interestingly, CD155 and CD112 bind to both the inhibitory receptor T-cell immunoreceptor with Ig and immunoreceptor tyrosine-based inhibitory (ITIM) domains (TIGIT) and the activating receptor DNAX accessory molecule-1 (DNAM-1) [9].

Histone mutations involving the substitution of lysine 27 (K27) with methionine (M) in H3.3/H3.1 (H3K27M) are the molecular hallmark of DMG [10]. Functional studies revealed that H3K27M mutation drives or accelerates DMG growth, suggesting that epigenetic dysregulation promotes DMG tumorigenesis [11, 12]. In support of this hypothesis, previous studies have shown that small-molecule inhibitors of histone deacetylases (HDACs) inhibit DMG growth in vitro and in vivo [13]. HDACs and histone acetyltransferases (HATs) maintain the dynamic balance of histone acetylation, regulating chromatin accessibility and downstream gene transcription [14]. The activity of HDACs on nonhistone proteins is also a key aspect of HDAC function [15]. However, the HDAC inhibitor (HDACi) panobinostat, a pan-HDACi, is used as a monotherapy for this aggressive tumor, but DMG cells eventually develop drug resistance [13]. Additionally, a recent phase I clinical trial revealed that panobinostat monotherapy yielded no significant clinical benefit in DMG patients [16]. The high failure rate of monotherapies and clinical trials suggests that combinatorial approaches may be necessary to improve patient outcomes.

Furthermore, studies have shown that HDAC inhibition can alter the tumor cell phenotype, increase the expression of NK-activating ligands, and promote NK cell-mediated killing, suggesting a potential strategy for combination treatment [17–25]. Moreover, immune checkpoint blockade (ICB) therapies have shown substantial clinical benefit in various cancers [26]. Systemic neutralization of NKG2A has been shown to induce a strong antitumor response in preclinical mouse studies by enhancing NK and/or CD8+ T-cell effector functions, which are further enhanced when NKG2A blockade is combined with anti-PD-(L)1 therapy [27]. The results of our preliminary experiment indicate that HDACis can modulate the expression of HLA-E. Therefore, combining HDACis with NK cell therapy and HLA-E–NKG2A axis blockage might be considered an effective strategy for treating DMG.

In conclusion, our study will investigate the use of HDACis in combination with NK cell therapies for DMG treatment and elucidate the mechanism by which HDACis increase HLA-E expression, thereby affecting HLA-E–NKG2A axis.

## Methods

### Cell culture and reagents

Primary human DMG cell lines (TT150630, TT190326, TT150714, TT150728, TT210305, DIPG17 and TT170720) were obtained and maintained as previously described [28]. DMG cells were cultured in 1% matrigel (Coning, Cat# 356243)-coated plates at 37 °C for 4 h, and then maintained in DMEM (Gibco, Cat# C11995500BT) supplemented with N2 (10 ng/mL; Gibco, Cat# 17502048), B27 (20 ng/mL; Gibco, Cat# 17504044), EGF (20 ng/mL; Scintol, Cat# SC102), bFGF (20 ng/mL; Scintol, Cat# SC107), ITS (Scintol, Cat# SC25800) and 1% Pen-Strep Amphotericin B (Gibco, Cat# 15140122). Human NK-92MI (HyCyte, Cat# TCH-C293) cells were maintained in MEM-α media without nucleosides (Gibco, Cat# 12561056) supplemented with 12.5% Fetal Bovine Serum (VivaCell, Cat# C04001-500), 12.5% Horse Serum (VivaCell, Cat# C2510-0500), 0.02 mM folic acid (MCE, Cat# HY-16637), 0.2 mM inositol (Sigma, Cat# I5125), 0.1 nM 2-mercaptoethanol (Sigma, Cat# M6250) and 1% Pen-Strep Amphotericin B (Gibco, Cat# 15140122). All cells were cultured at 37 °C with 5% CO2. DMG cells were digested with Accutase (Stemcell, Cat# 07921) and transferred to new plates every 3 days. The cell lines used in this study were tested for mycoplasma contamination via the Mycoplasma Detector and tested negative (Vazyme, Cat# D101-02). The respective cell line sources authenticated the cells.

### Cell viability assay

Cell viability was measured using a Cell Counting Kit-8 (CCK-8) assay (Biosharp, Cat# BS350A). 2 × 10^3^ DMG cells were seeded in 96-well plates and incubated overnight to allow attachment. Subsequently, cells were treated with various concentrations of MS-275 (MCE, Cat# HY-12163). Cell viability was evaluated using the CCK-8 assay. Absorbance was measured at 450 nm using a Synergy H1 MFD spectrophotometer (BioTek).

### EdU cell proliferation assay

Cell proliferation in response to MS-275 or DMSO treatment was assessed using an EdU assay, following the manufacturer’s instructions. About 1 × 10^5^ cells were seeded in 24-well plates and maintained for 48 h before the assay. 500 µL of 10 µM EdU reagent (Beyotime, Cat# C0071S) was added to each well and incubated for 2 h to label the cells. After washing with PBS, cells were fixed with 4% paraformaldehyde (Biosharp, Cat# BL539A) for 10 min, permeabilized with 0.3% Triton X-100 (Solarbio, Cat# T8200) for 10 min, and incubated with the click-reaction reagent for 30 min at room temperature in the dark. In all, 1 × Hoechst33342 reagent was used to counterstain the nucleus. Finally, cells were visualized using an inverted microscope (Zeiss, Axio Observer ZI, USA).

### Cell cycle assay

The Cell Cycle and Apoptosis Analysis Kit (Biosharp, Cat# BL114A) was used to assess cell cycle of DMG cells treated with MS-275 or DMSO. Cells were washed with cold PBS, fixed in 70% ethanol, and stored at 4 °C overnight for subsequent cell cycle analysis. Fixed cells were washed once with PBS and re-suspended in 1 mL of PI staining reagent (50 mg/mL propidium iodide and 1 mg/mL RNAse in sodium citrate buffer, pH 7.4). The samples were incubated in the dark for 30 min before cell cycle analysis. Cell cycle distribution was measured using a flow cytometer (BD Accuri C6 Plus), and quantified using FlowJo 10.6.2 Software. The percentages of cells in the G1, S, and G2 phases were calculated.

### Apoptosis assay

Apoptosis was analyzed using the BD Pharmingen FITC Annexin V Apoptosis Detection Kit (BD Biosciences, Cat# 556547) following the manufacturer’s instructions. Briefly, DMG cells were seeded into 6-well plates incubated with 1μM MS-275. After 48 h, the cells were harvested and stained with propidium iodide (PI) and FITC Annexin V solution. The percentage of apoptotic cells was analyzed using a BD Accuri C6 Plus flow cytometer, and data were processed with FlowJo 10.6.2 software.

### Flow cytometric analysis

Cells were washed with cell staining buffer (BioLegend, Cat# 420201), and incubated with Human TruStain FcX (BioLegend, Cat# 422301) for 15 min at room temperature. Cells were then incubated with anti-HLA-E (BioLegend, Cat# 342604), anti-CD107a (BioLegend, Cat# 328620), or isotype control antibodies (BioLegend, Cat# 400112) at 4 °C for 20 min. Signals were detected using a flow cytometer (BD Accuri C6 Plus, USA), and data were analyzed with FlowJo 10.6.2 Software.

### Quantitative real-time PCR assay

DMG cells were treated with MS-275 before total RNA isolation using RNeasy Mini Kit (QIAGEN, Cat# 74104) following the manufacturer's instructions. cDNA was synthesized using the S6 Super qPCR RT Kit (Scintol, Cat# S6166). Quantitative PCR (qPCR) was performed on a Quant Studio 5 (Applied Biosystems) using SYBR Green (Applied Biosystems, Cat# 4367659). The amplification program was as follows: initial denaturation step at 95 °C for 30 s, followed by 40 cycles at 95 °C for 15 s and 60 °C for 60 s. All assays were performed in triplicate, and average fold changes were calculated based on ACTB mRNA levels in the threshold cycle. The relative expression levels for each gene were determined via the 2 − ^∆∆CT^ method. The primer sequences are listed in Supplemental Table 1.

### Western blot analysis

Tumor cells were lysed using nondenaturing lysis buffer (Applygen, Cat# C1050) with 1% protease inhibitor cocktail (Solarbio, Cat# P6730) and 1% phosphatase inhibitor (Solarbio, Cat# P1260). Lysates were separated via SDS-PAGE (Bio-Rad) and transferred to PVDF membranes (Merck Millipore Ltd., Cat# IPVH00010). The membranes were probed for STAT3 (CST, Cat# 9139), ac-STAT3 K685 (CST, Cat# 2523), p-STAT3 S727 (CST, Cat# 94994), p-STAT3 Y705 (CST, Cat# 9145), HLA-E (proteintech, Cat# 66530-1-Ig), and β-actin (Abcam, Cat# ab20272). Protein bands were visualized using enhanced chemiluminescence reagents (NCM Biotech, Cat# P10300) on an Amersham Imager 600 (GE) and analyzed with ImageJ software (https://imagej.nih.gov/ij/download.html).

### Luciferase-based cytotoxicity

A luciferase-based cytotoxicity assay was performed as previously described [29]. Co-incubation experiments were conducted to assess the cytotoxicity of NK-92MI cells against luciferase-engineered DMG cells. Specifically, 10^4^ DMG cells in 50μL were co-cultured with 50μL of NK-92MI cells at different effector-to-target (E:T) ratios of 1:1, 2:1, 5:1, 10:1 and 20:1 for 4 h at 37 °C in a 5% CO2 atmosphere, using a 96-well plate. Tiragolumab (MCE, Cat# HY-P9986) and Monalizumab (MCE, Cat# HY-P99032) were used to block NK inhibitory receptor TIGIT and NKG2A, respectively. Briefly, NK-92MI cells were preincubated with Tiragolumab (50 μg/ml) or Monalizumab (50 μg/ml) for 1 h at 37℃ and 5% CO2 then co-cultured with DMG cells at the indicated E:T ratios. After incubation, Bright-Lite® Luciferase Assay System (Vazyme, Cat# DD1204) was used to detect the bioluminescence of survival cell according to the manufacturer’s protocol. A 100 μL mixture of Bright-Lite® Luciferase buffer and substrate was added to each well, and triplicate wells were used for each experiment. Bioluminescence was measured with a microplate reader, and the cytotoxicity (%) was calculated using the formula ((FC - FE)/FC) × 100, where FC represents the bioluminescence value of the control group and FE represents the bioluminescence value of the experimental group.

### Calcein AM based cytotoxicity

A calcein AM cytotoxicity assay was performed as previously described [30]. To stain DMG cells, 1 μM of calcein AM (MCE, Cat# HY-D0041) was added to the medium (1 × 10^6^ cells/ml) and the cells were incubated for 60 min at room temperature. DMG cells were washed once with PBS and re-suspended in complete medium at a concentration of 1 × 10^4^ cells/ml. NK-92MI cells were plated in triplicate in 96-well plates at a concentration of 1 × 10^4^ cells/ml to achieve a 1:1 ratio. The plates were incubated at 37 °C in CO2 for 4 h. After incubation, fluorescent images were captured using a Cytation5 microscope.

### Chromatin immunoprecipitation with qPCR (ChIP–qPCR)

Cells were crosslinked with 1% formaldehyde (final concentration) for 10 min at room temperature by inverting the flasks, and then quenched with 0.125 M glycine for 5 min. The cell pellets were repeatedly washed with PBS and stored at −80°C. The pellets were lysed in lysis buffer (50 mM HEPES, 150 mM NaCl, 1 mM EDTA, 0.1% SDS, 0.1% sodium deoxycholate, 1% Triton X- 100, and a protease inhibitor cocktail) for 10 min. After centrifugation, the supernatant was discarded, and the pellet was lysed in lysis buffer and subjected to sonication. Sheared chromatin was incubated overnight with ChIP-grade antibodies specific to STAT3 (CST, Cat# 9139S) or IgG (CST, Cat# 2729P) bound to PierceTM Protein A/G Agarose Beads (Thermo Fisher Scientific, Inc.), followed by elution and reverse cross-linking at 65 °C. TE buffer (10 mMTris-Hcl, 1 mM EDTA) was added in DNA elution buffer followed by RNase treatment (0.5 mg/mL) at 37 °C for 30 min and by proteinase K treatment (0.3 mg/mL) at 51 °C for 1 h, and the DNA was isolated and purified subsequently. The ChIP-enriched DNA was then decrosslinked and analyzed by real-time PCR. The primers used to amplify specific regions of HLA-E genes were as follows: human HLA-E- 1 forward, 5'- GCAGAGATACCGAAACCTAAAA − 3', human HLA-E- 1 reverse, 5'- AGGAAGAAGGACACATGACCA − 3'; human HLA-E- 2 forward, 5'- GCAGAGATACCGAAACCTA − 3', human HLA-E- 2 reverse, 5'- GAAACCCGACACCCATT − 3'; human HLA-E- 3 forward, 5'- ACTGCTGATTGCTGGGA − 3', human HLA-E- 3 reverse, 5'- ATAGTCGGGAGTCGTGG − 3'.

### Lentiviral infection and generation of DMG stable cell lines

Lentiviral plasmids carrying shRNA targeting STAT3 were used to knock down the expression of STAT3, and lentiviruses were purchased from Genechem Biotechnology Co., Ltd. The shRNA sequences targeting STAT3 are listed in Supplemental Table 2. Cells were infected with lentiviruses for 72 h and then were selected with puromycin (Sigma, Cat# p8833).

### RNA sequencing

DMG cells were treated with 1 μM MS-275 before total RNA isolation. RNA libraries were prepared using the VAHTS Universal V6 RNA-seq Library Prep Kit for Illumina (Vazyme, Cat# NR604). The libraries were sequenced on the Illumina Nova Seq 6000. RNA-seq analysis was performed using HISAT2 (v 2.1.0) to align reads to the human reference genome (hg38). SAM files were converted to BAM format using Samtools (v. 1.3.1). Then transcript coverage of UCSC gene annotations was analyzed using featureCounts. The DESeq2 R package was used to identify differentially expressed genes between the two groups. The GSEA R package was used to perform GSEA enrichment analysis for KEGG and Hallmark gene sets.

### CUT&Tag assay

TT150630 cells, treated with 1 μM MS-275 or DMSO for 48 h, were mixed with NovoNGS® Concanavalin A-coated magnetic beads (Novoprotein, Cat# N251). Resuspend the cells in ice-cold antibody buffer and add 1 µg anti-H3K27ac antibody (Abcam, Cat# ab177178) to each sample. Mix the secondary antibody at 1:100 in Dig-wash buffer, gently vortexing to dislodge the beads from the sides. Mix the pA-Tn5 adapter complex in Dig-300 buffer to a final concentration of 1:250 for 100 µL per sample. Add 100 µL of the pA-Tn5 mix, gently vortexing to dislodge most or all of the beads. After a quick spin, place the tubes on a magnetic stand to clear and remove the liquid. Add 300 µL of tagmentation buffer, then proceed to DNA extraction. The rest of the CUT&Tag library preparation was performed using the TruePrep DNA Library Prep Kit V2 for Illumina (Vazyme, Cat# TD503). The qualified libraries were sequenced on the Illumina NovaSeq 6000.

### CUT&Tag analysis

The resulting FASTQ files were trimmed with TrimGalore (v0.5.0) and aligned to hg38 using Bowtie2 (v 2.3.3). PCR duplicates were removed with PicardTools. The SAM alignment files were sorted with Samtools (v1.3.1). Peak calling was performed using MACS2 (v. 2.1.1.20160309). Peak annotation and motif analysis were conducted with Homer2 (v4.1.5). To visualize CUT&Tag-seq data, aligned BAMs from replicate experiments were combined using the Samtool merge function in Samtools. Bigwig files were generated from bam files using the bamCoverage function in deepTools. Track diagrams were generated using Integrated Genomics Viewer (IGV) for visualization. The peak by sample count matrix was obtained by counting reads overlapping with each peak. The count matrix was loaded into DESeq2 for differential analysis. Volcano plots were generated with ggplot2, showing log10-transformed *p*-values on the y-axis and log2 fold-change on the x-axis.

### Orthotopic xenograft mouse models

About 2 × 10^5^ luciferase-engineered TT150630 cell lines were suspended in 5 μL PBS and injected into the brainstem of NSG mice as previously describe [28]. After 7 days, fluorescence imaging of the mice was carried out to determine the success of the tumor inoculation. Then the mice successfully inoculated with tumors were divided into different groups (n = 5). MS-275 (10 mg/kg in a final formulation in 2% DMSO) was administered by intraperitonally daily for 2 weeks. For combination therapy, MS-275 was administered for two days before being combined with intratumoral NK-92MI injections (2 × 10^6^), with or without Monalizumab (50 μg, MCE, Cat# HY-P99032) or an isotype control antibody (50 μg, MCE, Cat# HY-P99003).

The tumor burdens were monitored once a week by bioluminescence imaging using an IVIS Spectrum (PerkinElmer) after injected with 150 μL of D-Luciferin. The fluorescence intensity was quantified via Living Image, a software program provided by the same manufacturer. The major organs were fixed in 10% formalin, embedded in paraffin, and sliced into 5 μm sections for H&E staining.

### Statistical analysis

Results were analyzed using a two-tailed unpaired Student's t-test or one-way ANOVA in GraphPad Prism 9.0 (GraphPad Software, Inc.). Quantitative data are presented as the mean ± standard deviation (SD) from at least three samples or experiments per data point. For the in vivo assay, survival analysis was performed using the Kaplan–Meier method, and differences between groups were calculated using the Log-rank test. A *p*-value of < 0.05 was considered the significance threshold for all analyses. Significance levels were indicated as follows: *p < 0.05, **p < 0.01, ***p < 0.001.

## Results

### Inhibition of HDACs by MS-275 suppressed the proliferation of DMG cells in vitro and in vivo

To explore the correlation between HDAC gene expression and survival in brainstem glioma (BSG) patients, we conducted survival analysis of a cohort of 98 BSG patients (EGA Archive: EGAS00001004341) [31]. Our results revealed a significant correlation between elevated expression levels of HDAC1, HDAC2, and HDAC4 and poor survival in BSG patients (Fig. S1A). Among these 98 BSG patients, 58 (59.2%) were diagnosed with DMGs with H3K27M mutation (considered the DMG group), 38 (38.8%) were H3K27 wild-type, and 2 had an unknown H3 mutation status. Further stratified analysis within the DMG subgroup indicated that patients with elevated expression of HDAC2 exhibited significantly worse survival outcomes (Fig. S1B). In contrast, among patients with H3K27 wild-type BSGs, HDAC gene expression levels had no significant impact on survival outcomes (Fig. S1C). To further investigate the potential role of HDAC genes in promoting DMG progression, we analyzed a CRISPR/Cas9 loss-of-function screening dataset for 32 DMG cell lines [32, 33]. Among the 8 genes mapping to the HDAC family (excluding HDAC7, 10, and 11), HDAC2 and HDAC3 demonstrated strong genetic dependencies, indicating a critical role in supporting DMG cell growth and proliferation (Fig. S2A). On the basis of these findings, we next aimed to advance our study by evaluating the therapeutic potential of MS-275, a class I HDACi that targets HDAC1, HDAC2, and HDAC3, as a prospective treatment strategy for DMG.

**Fig. 1:**
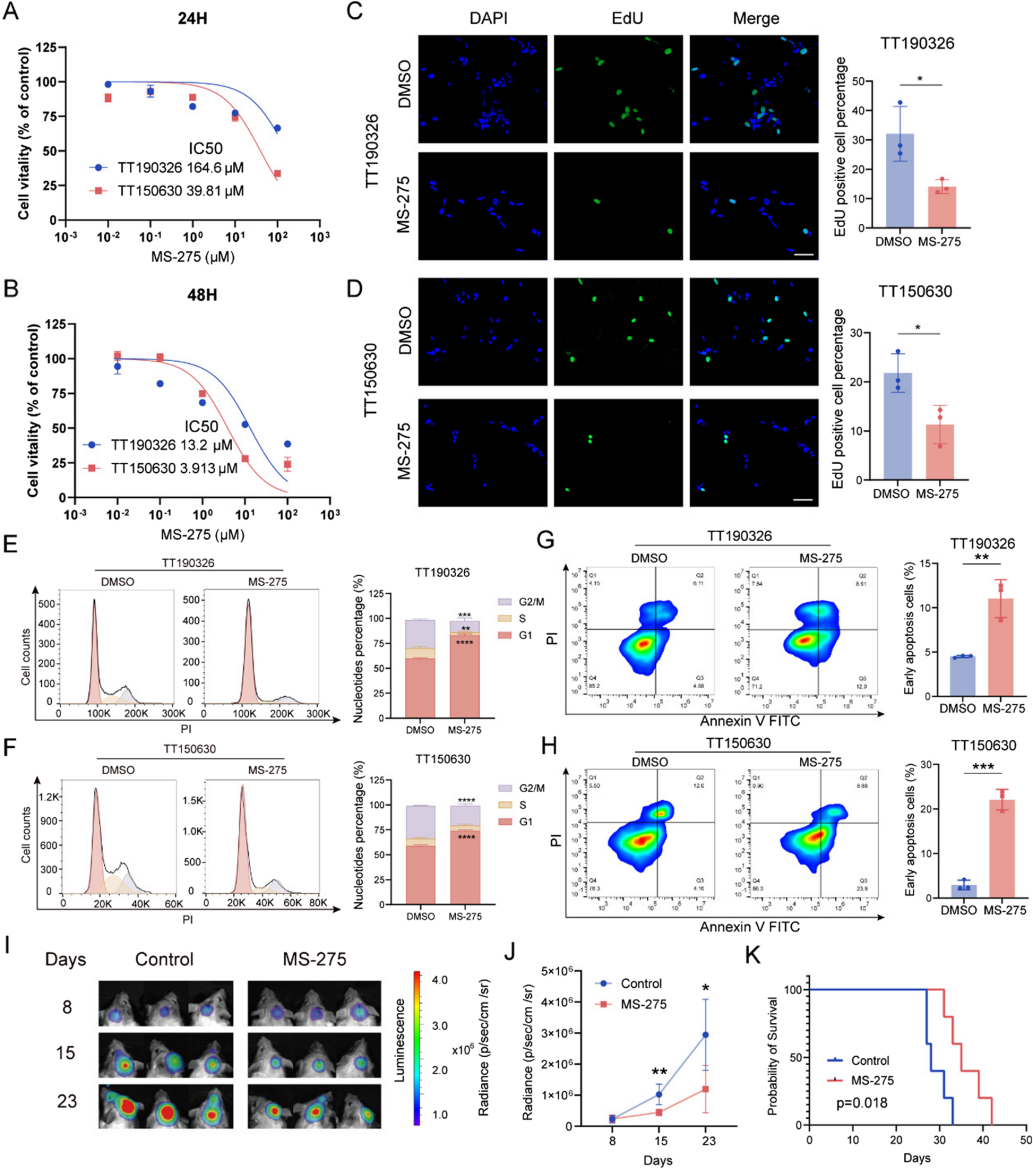
The inhibitory effects of MS-275 on DMG cells in vitro and in vivo. **A**-**B** The inhibitory effects of MS-275 on TT190326 and TT150630 cells were demonstrated via CCK-8 assays. The cells were treated with various concentrations of MS-275 (0.01–100 µM) for 24 and 48 h. **C**-**D** Representative immunofluorescence image of EdU-stained TT150630 and TT190326 cells after treatment with 1 μM MS-275 or vehicle for 2 days (left). Average percentage of EdU-positive TT150630 or TT190326 cells after treatment with DMSO or MS-275 (right). Scale bars, 50 μm. Statistical significance was assessed via Student’s t test, with *p < 0.05. **E**–**F** Distribution (left) and quantitation (right) of the cell cycle in TT150630 or TT190326 cells treated with DMSO or 1 μM MS-275 for 2 days. Statistical significance was assessed via Student’s t test, with **p < 0.01, ***p < 0.001, and ****p < 0.0001. **G**-**H** Flow cytometry analysis of apoptotic TT150630 or TT190326 cells after treatment with DMSO or 1 μM MS-275 for 2 days (left). The percentages of Annexin V-positive and PI-negative (early apoptotic) cells were quantified with FlowJo software (right). Statistical significance was assessed via Student’s t test, with **p < 0.01 and ***p < 0.001. **I** Representative bioluminescence images (BLIs) of mice xenografted with TT150630-luciferase cells and treated with or without MS-275 (*n* = 5/group). **J** Quantification of the BLI signal in (**I**). Statistical significance was assessed via Student’s t test, with *p < 0.05 and **p < 0.01. **K** Kaplan‒Meier survival curves for mice xenografted with TT150630-luciferase cells and treated with or without MS-275 (*n* = 5/group). *P* values were calculated via the log-rank test

In these subsequent experiments, we focused on assessing the therapeutic efficacy of MS-275 in the treatment of DMG. Initially, we assessed the inhibitory effects of MS-275 on cell viability across different primary DMG cell lines at various concentrations and time points. The results revealed a dose-dependent reduction in cell viability following MS-275 treatment (Fig. 1A, B and Fig. S3A, S3B). We also performed EdU assays to assess the antiproliferative effect of MS-275 on DMG cells. The results revealed that treatment with 1μM MS-275 for 2 days significantly reduced the number of EdU-positive TT150630 and TT190326 cells, indicating that MS-275 inhibits DMG cell proliferation (Fig. 1C and D). Next, we performed flow cytometry analysis to investigate the impact of MS-275 on cell cycle progression and apoptosis in TT150630 and TT190326 cells. In both cell lines, MS-275 treatment induced G1-phase arrest, thereby impeding progression to the S phase and effectively inhibiting cell growth in vitro (Fig. 1E and F). Moreover, FACS analysis via Annexin V staining revealed an increased proportion of early apoptotic cells among MS-275-treated cells for both cell lines, indicating that MS-275 also promotes apoptosis (Fig. 1G and H).

To further elucidate the molecular mechanisms underlying the effects of MS-275 on DMG cells, we performed RNA sequencing (RNA-seq) to analyze the transcriptomes of TT150630 and TT190326 cells treated with 1μM MS-275 for 48 h. Consistent with our previous findings, MS-275 treatment resulted in significant downregulation of positive cell cycle regulators (e.g., CCNA1 and E2F1) and upregulation of negative cell cycle regulators (e.g., CDKN2B and CDKN1A), suggesting a shift toward cell cycle arrest. Moreover, MS-275 treatment led to decreased expression of apoptosis-inhibiting genes, such as BCL2, and increased expression of proapoptotic genes, such as BAX, further supporting its role in promoting apoptosis in DMG cells (Fig. S4A and S4B). Notably, a similar trend of expression was also observed for these genes in MS-275-treated SU-DIPG XIII cells, as reported in the GSE110572 dataset [34] (Fig. S4C). We subsequently used a mouse orthotopic xenograft model with TT150630-Luc cells to assess the efficacy of HDAC inhibition in vivo. Tumor-bearing mice were randomly assigned to (i) the control group or (ii) the MS-275 group. The treated group received an intraperitoneal injection of MS-275 (10 mg/kg) for two weeks. The tumor volume of NSG mice was assessed via IVIS at different time points (8, 15, and 23 days after inoculation with tumor cells) (Fig. 1I). We observed reduced tumor growth in the MS-275 group compared with the control group after two weeks of MS-275 treatment (Fig. 1J). Moreover, analysis of Kaplan‒Meier survival curves revealed that MS-275 treatment significantly prolonged the survival of the mice (*p* = 0.018) (Fig. 1K). In addition, pathomorphological analysis of heart, liver, spleen, lung, and kidney samples from the MS-275 group revealed no observable signs of organ toxicity (Fig. S5A).

### MS-275 upregulated the NK cell-mediated cytotoxicity pathway and increases the expression of NK cell ligands in DMG

**Fig. 2:**
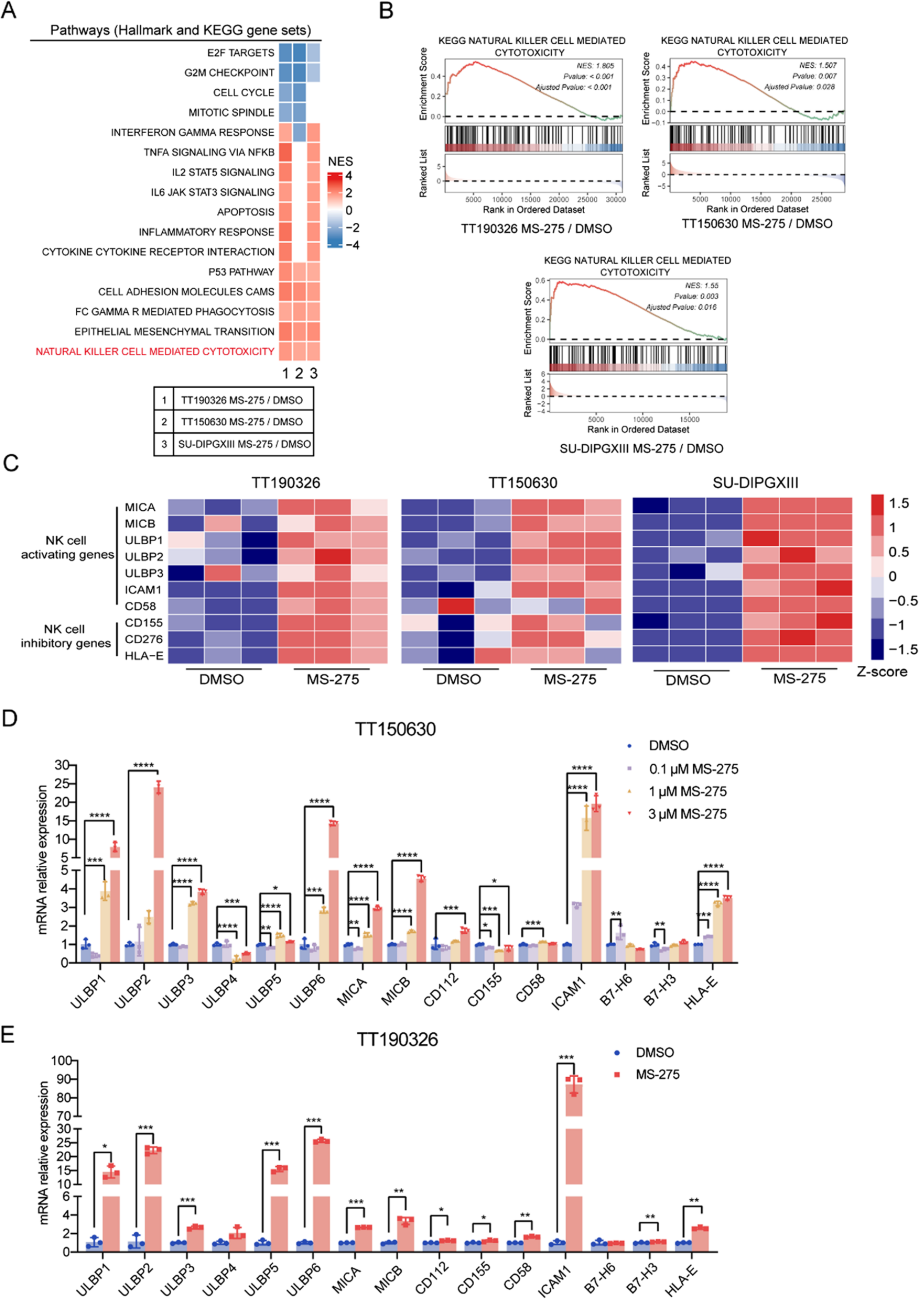
MS-275 activates NK cell-mediated cytotoxic pathways and modulates NK cell ligand expression. **A** Normalized enrichment scores (NESs) for the indicated KEGG and hallmark gene sets (MSigDB) that were enriched in MS-275-treated TT150630, TT190326, and SU-DIPGXIII cells relative to control cells (adjusted *p* < 0.05) in the gene set enrichment analysis (GSEA) (performed with the GSEA R package). The lack of significant changes (*p* > 0.05) colored white. **B** GSEA analysis using the “KEGG nature killer cell-mediated cytotoxicity” gene set to compare MS-275-treated and control DMG cells (TT190326, TT150630, and SU-DIPGXIII). **C** Heatmap of NK cell ligand genes from RNA-seq transcriptome analysis of TT150630, TT190326, and SU-DIPGXIII cells. **D** RT‒qPCR analysis of the relative expression of NK cell ligands in TT150630 cells treated with different concentrations of MS-275 (0.1 µM, 1 µM, or 3 µM) for 2 days. Statistical significance was assessed via one-way ANOVA with *p < 0.05, **p < 0.01, ***p < 0.001, and ****p < 0.0001. **E** RT‒qPCR analysis of the relative expression of NK cell ligands in the indicated DMG cells treated with 1µM MS-275 for 2 days. Statistical significance was assessed via Student’s t test, with *p < 0.05, **p < 0.01, ***p < 0.001, and ****p < 0.0001

To further evaluate the functional impact of MS-275 treatment on DMG cells, we conducted gene set enrichment analysis (GSEA) on the basis of transcriptomic data obtained from MS-275- or DMSO-treated DMG cells. Specifically, we aimed to identify the signaling pathways modulated by MS-275 treatment in this context. Enrichment analysis of the hallmark and KEGG gene sets revealed downregulation of cell cycle control pathways, which was consistent with the observed growth inhibition of MS-275-treated cells. Moreover, MS-275 treatment promoted immune-related pathways, particularly those associated with interferon (IFN)-dependent signaling and cell-intrinsic immune responses, suggesting that MS-275 may enhance immune surveillance in DMG cells (Fig. 2A). Notably, we detected significant upregulation of the NK cell-mediated killing pathway in TT150630, TT190326, and SUDIPG-XIII cells following MS-275 treatment (FDR < 0.05) (Fig. 2B).

We further assessed the expression of key tumor ligands for NK cells on the basis of our RNA-seq data. The results demonstrated a significant increase in both activating ligands (e.g., MICA, MICB, and ULBP1-3) and inhibitory ligands (e.g., CD276 and HLA-E) in MS-275-treated cells (Fig. 2C). To validate these findings, we treated TT150630 cells with various concentrations of MS-275 and measured the expression levels of these NK cell ligands. In line with our transcriptomic data, we observed a dose-dependent increase in the mRNA expression of ULBP1-3, ULBP6, MICA/B, CD112, ICAM1, and HLA-E following MS-275 treatment (Fig. 2D). These results were also consistent across multiple MS-275-treated DMG cell lines; six other DMG cell lines also exhibited similar upregulation of NK cell ligands (Fig. 2E and Fig. S6A-S6E). Collectively, these results demonstrated that MS-275 treatment induced the upregulation of several activating and inhibitory NK cell ligands that are crucial for NK cell-mediated cytotoxicity.

### Targeting the HLA-E–NKG2A axis in combination with MS-275 enhances NK cell-based immunotherapy against DMG

To assess whether NK cell-mediated cytotoxicity against diffuse midline glioma (DMG) cells is enhanced by the HDACi MS-275, we first assessed the cytotoxicity of NK-92MI cells against primary DMG cell lines via two different established methods [29, 30]. The first method involved quantifying bioluminescence in the culture media after coculturing luciferase-engineered DMG cells with NK-92MI cells at varying effector-to-target (E:T) ratios. As shown in Fig. S7A, the different DMG cell lines exhibited varying sensitivities to NK cell-mediated cytotoxicity, which increased with increasing E:T ratios. The second method involved a calcein AM-based fluorescence imaging assay. DMG cells were prelabeled with calcein AM dye and then incubated with NK-92MI cells at a 1:1 ratio (TT150630, TT190326, TT150714, TT210305, DIPG17 and TT170720) or a 5:1 ratio (TT150728). Subsequently, fluorescence imaging was used to quantify NK cell-mediated cytotoxicity. The results demonstrated reduced fluorescence in DMG cells, indicating cell death, following coculture with NK-92MI cells (Fig. S7B).

**Fig. 3:**
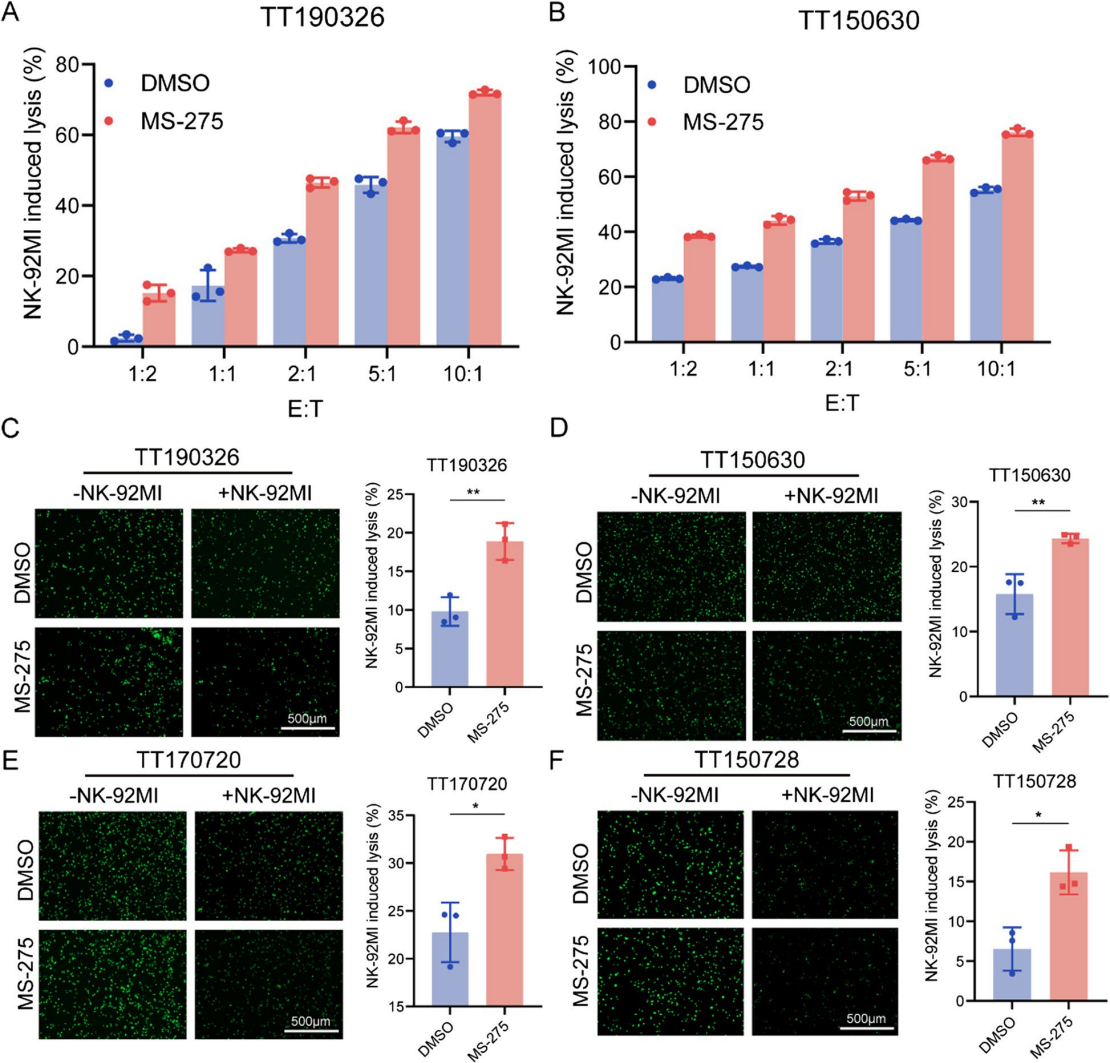
MS-275 enhances NK cell-mediated cytotoxicity against DMG cells. **A**-**B** Luciferase-engineered TT150630 and TT190326 cell lines were treated with DMSO or 1µM MS-275 for 2 days and cocultured with NK-92MI cells at the indicated E:T ratios. After 4 h of incubation, bioluminescence was measured, and NK cell-induced cytotoxicity (%) was calculated and plotted. **C**-**F** DMG cells were treated with MS-275 (1µM) for 2 days, stained with calcein AM, and cocultured with NK-92MI cells in 96-well plates at a 1:1 ratio (TT190326, TT150630 and TT170720) or a 5:1 ratio (TT150728). After incubation for 4 h, fluorescence images were captured using an inverted microscope. Images of DMG cell lines showing a reduction in fluorescence (indicating cell death) are presented (left). Calcein AM-stained DMG cells without NK-92MI cells served as controls. The percentage (%) of cells undergoing NK cell-induced cytotoxicity was calculated and plotted (right). Statistical significance was assessed via Student’s t test, with *p < 0.05 and **p < 0.01. Scale bars, 500 μm

Furthermore, we treated DMG cells with the HDACi MS-275 to evaluate its impact on NK cell cytotoxicity via both the luciferase-based and calcein AM-based methods. Our findings demonstrated that MS-275 treatment significantly enhanced NK cell-induced cytotoxicity against DMG cells (Fig. 3A-F).

**Fig. 4:**
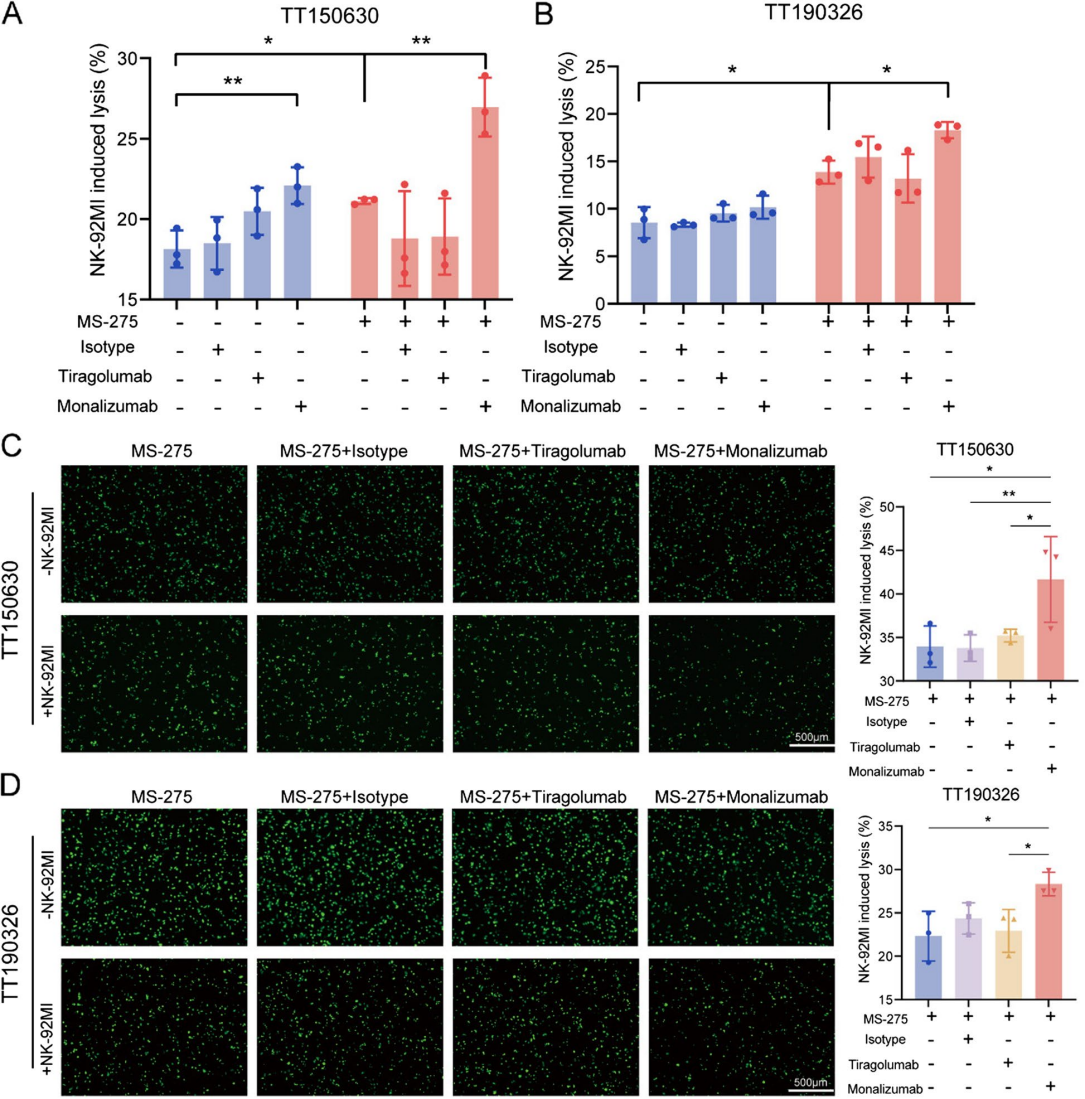
MS-275 enhances the efficacy of immunotherapies targeting the NKG2A–HLA-E axis in vitro. **A**-**B** Luciferase-engineered TT150630 and TT190326 cell lines were treated with DMSO or 1µM MS-275 for 2 days and cocultured with NK-92MI cells for 4 h at a 1:1 ratio. The NK-92MI cells were preincubated with control IgG (50 μg/ml), tiragolumab (50 μg/ml) or monalizumab (50 μg/ml) for 1 h at 37 °C before being cocultured with the tumor cells and the NK-92MI cells. The percentage (%) of cells undergoing NK cell-induced cytotoxicity was calculated and plotted. Statistical significance was assessed via one-way ANOVA, with *p < 0.05 and **p < 0.01. **C**-**D** TT190326 and TT150630 cells were treated with MS-275 (1µM) for 2 days, stained with calcein AM, and cocultured with NK-92MI cells for 4 h in 96-well plates at a 1:1 ratio. The NK-92MI cells were preincubated with control IgG (50 μg/ml), tiragolumab (50 μg/ml) or monalizumab (50 μg/ml) for 1 h at 37 °C before being cocultured with the tumor cells and the NK-92MI cells. Fluorescence images were captured using an inverted microscope (left). The percentage (%) of cells undergoing NK cell-induced cytotoxicity was calculated and plotted (right). Statistical significance was assessed via one-way ANOVA, with *p < 0.05 and **p < 0.01. Scale bars, 500 μm.

Considering that MS-275 treatment upregulated the expression of NK inhibitory ligands on the surface of DMG cells, we sought to determine whether blocking the inhibitory signaling mediated by these ligands could further enhance NK cell cytotoxicity. Our findings revealed increased expression of CD112, which interacts with the NK inhibitory receptor TIGIT, and HLA-E, which binds to NKG2A, in all MS-275-treated DMG cell lines. Furthermore, we preincubated NK-92MI cells with the anti-TIGIT antibody tiragolumab or the anti-NKG2A antibody monalizumab for 1 h at 37°C and assessed their cytotoxic efficacy against both untreated and MS-275-treated DMG cells. The results revealed a significant increase in NK cell-mediated tumor cell killing following MS-275 treatment, with NKG2A blockade by monalizumab further enhancing this effect; however, no additional benefit was observed with TIGIT blockade by tiragolumab (Fig. 4A-D). We also performed degranulation assays with NK cells. The results demonstrated that NK cells co-cultured with MS-275-treated DMG cells exhibited a significantly higher CD107a surface expression compared to those co-cultured with control DMG cells, suggesting that MS-275 treatment potentiated NK cell degranulation. Moreover, blockade of NKG2A further augmented this effect (Fig. S8A). Flow cytometry analysis confirmed that MS-275 treatment elevated surface HLA-E expression on DMG cells (Fig. S8B-S8H).

**Fig. 5:**
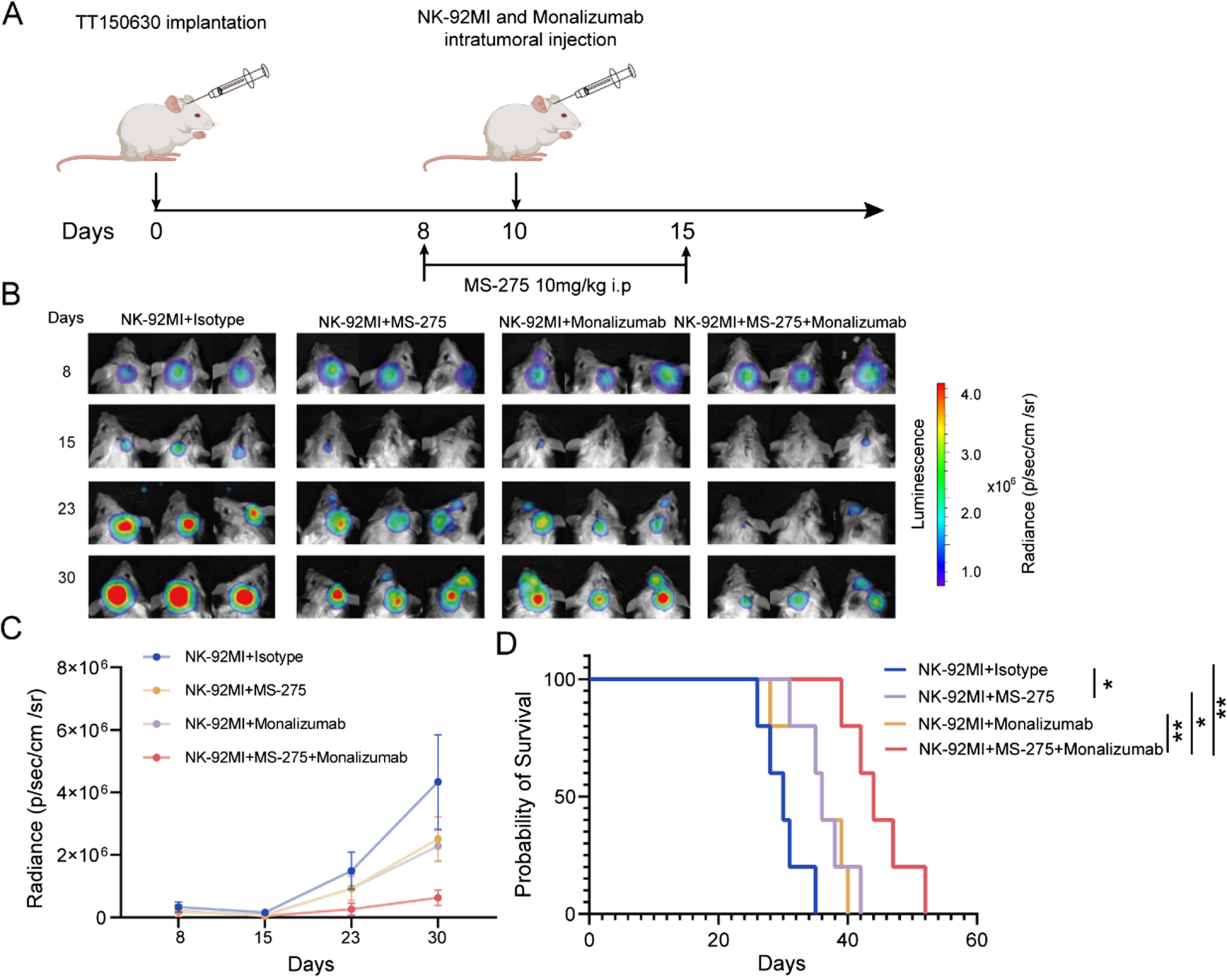
MS-275 enhances the efficacy of immunotherapies targeting the NKG2A–HLA-E axis in vivo. **A** Schematic diagram of the in vivo combination treatment in TT150630-bearing mice. The mice were treated with MS-275 (daily × 14 days, i.p.). MS-275 was administered for two days before being combined with intratumoral NK-92MI injections (2 × 10^6^), with either monalizumab (50 μg) or an isotype control antibody (50 μg). **B** Representative BLIs of orthotopic TT150630 tumors from the following 4 groups: (i) NK-92MI and isotype, (ii)NK-92MI and MS-275, (iii) NK-92MI and monalizumab and (iv) NK-92MI, MS-275 and monalizumab groups (*n* = 5/group). **C** Quantification of the BLI signal in (**B**). **D** Kaplan‒Meier survival curves for mice xenografted with TT150630-luciferase cells from each group are shown in (I) (*n* = 5/group). *P* values were calculated via the log-rank test.

We subsequently employed a mouse orthotopic xenograft model with TT150630-Luc cells and randomly assigned the mice into four groups: (i) NK-92MI and isotype; (ii) NK-92MI and MS-275; (iii) NK-92MI and monalizumab; and (iv) NK-92MI, MS-275, and monalizumab. MS-275 was administered for two days and then intratumoral NK-92MI injections, with or without monalizumab or an isotype control antibody (Fig. 5A). Compared with the other treatments, the combination of NK-92MI, MS-275, and monalizumab had the strongest inhibitory effect on tumor growth (Fig. 5B and C). Additionally, our data revealed that this combination significantly extended animal survival compared with that in the NK-92MI and isotype (*p* = 0.0018), NK-92MI and MS-275 (*p* = 0.0136), and NK-92MI and monalizumab (*p* = 0.0088) groups, as determined via Kaplan‒Meier analysis (Fig. 5D). Pathomorphological analysis of heart, liver, spleen, lung, and kidney specimens from all groups revealed no visible signs of toxicity (Fig. S8I).

### CUT&Tag analysis revealed H3K27ac-modified chromatin in TT150630 cells treated with MS-275

We further investigated the impact of the HDACi MS-275 on HLA-E expression, focusing on its epigenetic regulatory role. HDACis, such as MS-275, are known to modulate histone acetylation, thereby influencing gene transcription. To assess the effects of MS-275 on chromatin modifications, we conducted a CUT&Tag assay for H3K27ac in TT150630 cells.

**Fig. 6:**
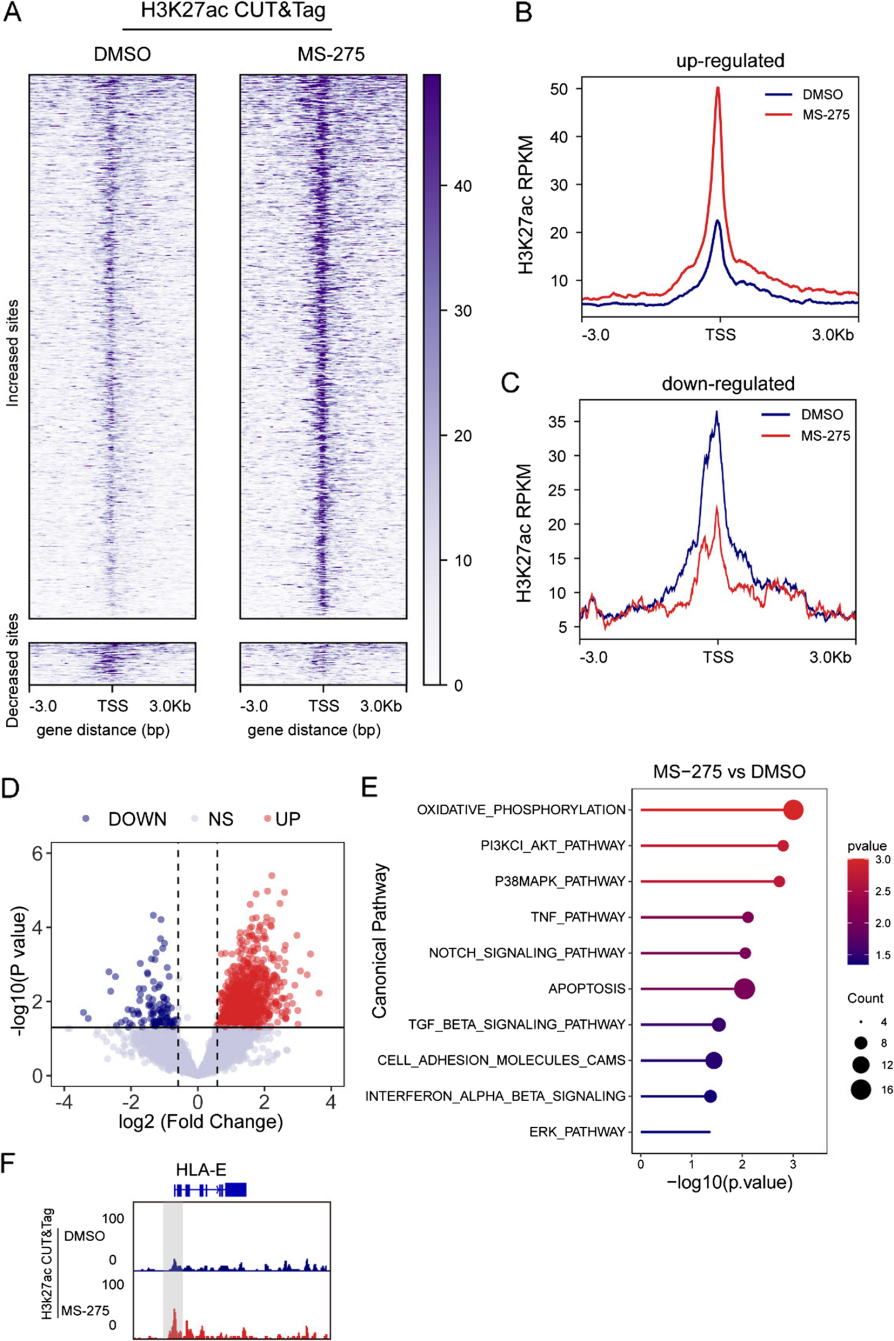
Epigenetic modulation and functional regulation by MS-275 in DMG cells. **A** CUT&Tag-seq profiles from TT150630 cells showing sites where treatment with MS-275 (1μM) for 2 days resulted in increased (top) or decreased (bottom) H3K27ac signals at the gene promoter (*p* < 0.05, |LFC|> 1.5) (*n* = 2). **B**-**C** Average H3K27ac signal corresponding to (**A**). **D** Volcano plots showing differentially expressed genes from H3K27ac CUT&Tag signals of TT150630 with or without MS-275 (1 μM) for 2 days. **E** Canonical pathway (MSigDB) enrichment analysis of 996 DEGs in MS-275-treated DMG cells compared with the DMSO-treated cells. **F** Integrated Genomics Viewer (IGV) screenshot showing results of H3K27ac CUT&Tag-seq in peaks at the genomic regions of HLA-E

Through analysis of the H3K27ac CUT&Tag data, we identified 922 genes exhibiting significantly increased H3K27ac levels at their promoters after MS-275 treatment (fold change > 1.5, *p* < 0.05), alongside 74 genes with reduced acetylation (fold change < − 1.5, *p* < 0.05) (Fig. 6A-D). Overrepresentation analysis (ORA) of these differentially expressed genes (DEGs) revealed that pathways associated with cell proliferation (e.g., the PI3K/AKT, P38 MAPK, and NOTCH signaling pathways), apoptosis, oxidative phosphorylation, and immune/inflammatory responses (e.g., the interferon alpha/beta signaling and TNF pathways) were altered upon MS-275 treatment (Fig. 6E). Consistent with our previous experimental findings and RNA-seq results, we detected increased H3K27ac signals in the promoter regions of apoptosis-related genes, such as *BAD* and *CASP7* (Fig. S9A), and decreased H3K27ac signals in the promoter regions of cell cycle regulatory genes, including *CDC45* and *CDC25 C* (Fig. S9B).

As anticipated, the H3K27ac CUT&Tag data revealed distinct transcriptional regulation between MS-275-treated diffuse midline glioma (DMG) cells and untreated controls (Fig. S5A and S5B). There were numerous regions with enhanced H3K27ac binding in MS-275-treated cells, indicating the activation of specific chromatin regions following drug exposure. Additionally, MS-275 treatment led to increased enrichment of H3K27ac in the promoter region of the HLA-E gene (Fig. 6F), further supporting its role in modulating HLA-E expression through epigenetic mechanisms.

### Unphosphorylated STAT3 binds the promoter region of HLA-E to drive its transcription

**Fig. 7:**
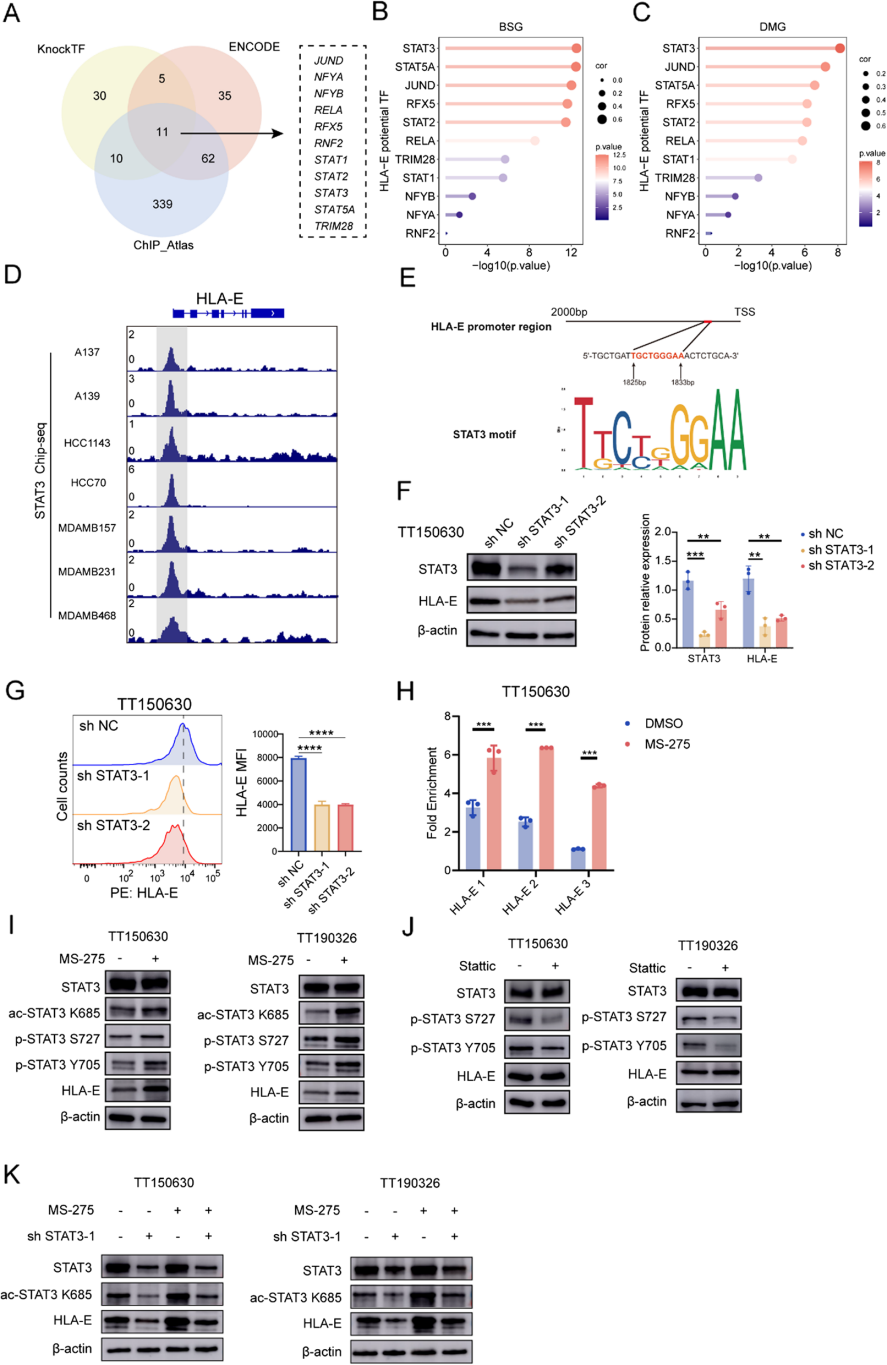
MS-275 enhances HLA-E expression through acetylation of STAT3. **A** A Venn diagram showing the analysis of the KnockTF, ENCODE, and ChIP-Atlas databases revealed 56, 113, and 422 HLA-E transcription factors, respectively. Intersection analysis identified 11 most likely HLA-E transcription factors, including JUND, NFYA, NFYB, RELA, RFX5, RNF2, STAT1, STAT2, STAT3, STAT5A and TRIM28. **B**-**C** Lollipop chart showing correlation analysis between 11 potential transcription factors (JUND, NFYA, NFYB, RELA, RFX5, RNF2, STAT1, STAT2, STAT3, STAT5A, and TRIM28) and HLA-E expression in BSG (**B**) or DMG (**C**) patients. The point size represents the correlation coefficient. **D** Integrated Genomics Viewer (IGV) screenshot showing the results of STAT3 ChIP-seq in peaks at the genomic regions of HLA-E. **E** Analysis of the JASPAR database showing the potential binding region of STAT3 to the HLA-E promoter. **F** The protein expression of HLA-E and total STAT3 in TT150630 cells expressing sh-NC or sh-STAT3 was detected via Western blotting. β-Actin was used as the internal control (left). The quantified results are presented in the plot (right). Statistical significance was assessed via one-way ANOVA with **p < 0.01 and ***p < 0.001. **G** HLA-E expression on the cell surface of TT150630 cells expressing sh-NC or sh-STAT3 was detected via flow cytometry. The quantification of the results is shown on the right. Statistical significance was assessed via one-way ANOVA with ****p < 0.0001. **H** TT150630 cells treated with MS-275 (1 μM) or DMSO for 2 days were analyzed for STAT3 recruitment to the HLA-E promoter via ChIP analysis. The ChIP results are shown as the fold change relative to the IgG control. Statistical significance was assessed via Student’s t test, with **p < 0.01 and ***p < 0.001. **I** The protein expression of HLA-E, total STAT3, p-STAT3 (S727), p-STAT3 (Y705), and ac-STAT3 (K685) in TT150630 and TT190326 cells treated with 1 μM MS-275 for 2 days was detected via Western blotting. β-Actin was used as the internal control. **J** The protein expression of HLA-E, total STAT3, p-STAT3 (S727), and p-STAT3 (Y705) in TT150630 and TT190326 cells treated with 1 μM Stattic for 2 days was detected via Western blotting. β-Actin was used as the internal control. **K** The protein expression of HLA-E, total STAT3, and ac-STAT3 (K685) in TT150630 cells or TT190326 cells expressing sh-NC or sh-STAT3 treated with DMOS or 1 μM MS-275 for 2 days was detected via Western blotting. β-Actin was used as the internal control

We subsequently assessed the impact of HDAC inhibition on transcription factors involved in the regulation of HLA-E expression. On the basis of comprehensive analysis using the KnockTF, ENCODE, and ChIP-Atlas databases [35–37], we identified 11 transcription factors (JUND, NFYA, NFYB, RELA, RFX5, RNF2, STAT1, STAT2, STAT3, STAT5 A and TRIM28) associated with the regulation of HLA-E expression (Fig. 7A). Correlation analysis revealed that STAT3 had the strongest positive correlation with HLA-E in both DMG (*R* = 0.643, *p* < 0.001) and BSG (*R* = 0.642, *p* < 0.001) (Fig. 7B-C and Fig. S11A-S11B). Furthermore, analysis of the GSE85579 dataset demonstrated that STAT3 can bind to the promoter region of HLA-E (Fig. 7D) [38]. The potential binding region of STAT3 on the HLA-E promoter was predicted via the JASPAR database (Fig. 7E) [39]. On the basis of the above evidence, we postulated that STAT3 directly regulates the expression of HLA-E as a transcription factor. Furthermore, we employed shRNAs to genetically target STAT3 in TT150630 and TT190326 cells. The results revealed a reduction in HLA-E protein levels in cells with STAT3 knockdown (Fig. 7F and Fig. S12A). This observation was further validated by flow cytometry analysis of TT150360 and TT190326 cells expressing shSTAT3 (Fig. 7G and Fig. S12B).

We subsequently performed chromatin immunoprecipitation (ChIP) assays to assess the recruitment of STAT3 to HLA-E promoters in TT150630 cells treated with MS- 275. Our results demonstrated significant enrichment of STAT3 at the HLA-E promoter, which was further enhanced by MS-275 treatment (Fig. 7H).

Additionally, we investigated the mechanism by which HDAC inhibition upregulates HLA-E protein expression via STAT3. Considering the potential impact of HDAC inhibition on gene expression, we hypothesized that treatment with the HDACi MS-275 upregulates STAT3 expression, subsequently leading to increased HLA-E levels. In support of our hypothesis, CUT&Tag data revealed elevated H3K27ac signals at the STAT3 promoter in MS-275-treated TT150630 cells, suggesting that MS-275 enhances chromatin accessibility at the STAT3 locus (Fig. S7C). Moreover, real-time PCR analysis revealed that MS-275 treatment resulted in increased mRNA expression of both STAT3 and HLA-E in TT190326 and TT150630 cells (Fig. S7D). However, Western blot analysis revealed an increase in HLA-E protein expression but not in STAT3 protein levels upon MS-275 treatment in DMG cells (Fig. 7I and Fig. S12E). We further investigated the effect of MS-275 on STAT3 phosphorylation, a posttranslational modification that activates STAT3. Western blot analysis revealed that HDAC inhibition induced STAT3 phosphorylation at Y705 and S727 following MS-275 treatment in DMG cells (Fig. 7I and Fig. S12E). To validate the role of phosphorylated STAT3 in regulating HLA-E, we assessed the expression of HLA-E in TT150630 and TT193026 cells treated with Stattic, a STAT3 phosphorylation inhibitor. However, Stattic significantly inhibited STAT3 phosphorylation at Y705 and S727 but did not affect HLA-E expression (Fig. 7J and Fig. S12F).

Considering the influence of HDACs on nonhistone proteins, we hypothesized that the HDACi MS-275 might facilitate STAT3 acetylation to increase HLA-E expression. In support of this hypothesis, previous studies have shown that STAT3 acetylation at K685 enhances the transcriptional activity of nonphosphorylated STAT3 [40]. Therefore, we first assessed the impact of MS-275 treatment on STAT3 acetylation at K685 in DMG cells. The results revealed that treatment with MS-275 significantly increased STAT3 acetylation at K685 in TT150630 and TT190326 cells (Fig. 7I and Fig. S12E). To further investigate whether STAT3 acetylation mediates HLA-E expression, we treated STAT3-knockdown cells with 1 µM of the HDACi MS-275 for 2 days. Our findings demonstrated that STAT3 knockdown suppressed acetylation at K685 and decreased HLA-E expression. Furthermore, MS-275 treatment partially restored both STAT3 acetylation at the K685 site and HLA-E expression in STAT3-knockdown cells (Fig. 7K and Fig. S12G).

In conclusion, our results indicate that the upregulation of HLA-E expression induced by the HDACi MS-275 may be attributed to its modulation of K685 acetylation on STAT3, which potentially acts as a transcription factor for HLA-E (Fig. S12H).

## Discussion

Despite decades of preclinical and clinical studies, DMG remains incurable. DMG is characterized by a histone H3 mutation (H3K27M) that alters chromatin accessibility and gene expression, contributing to its malignant growth [11]. The aberrant chromatin regulation induced by H3K27M mutation suggests that epigenetic therapy is a promising approach for treating DMG. Previous studies have shown that epigenetic regulators such as BRD4, EZH2, and HDACs are effective targets of DMG, and the corresponding small molecule inhibitors have demonstrated potent antitumor effects in vitro and in vivo [13, 41, 42]. Among these epigenetic inhibitors, panobinostat, a pan-HDACi that was approved by the FDA for adults with multiple myeloma, is considered a promising therapy for diffuse intrinsic pontine glioma (DIPG) [13]. However, owing to poor drug tolerability and limited penetration into the central nervous system, oral panobinostat has shown no significant clinical benefit in DMG patients [16]. Conversely, repeated delivery of panobinostat via convection-enhanced delivery (CED) achieves therapeutic drug concentrations and prolongs the survival of DMG patients [43]. Therefore, an HDACi that is durable and crosses the blood‒brain barrier is needed to replace panobinostat. In this study, survival analysis of 58 DMG patients revealed that high HDAC2 expression is significantly linked to a poor prognosis. A public CRISPR/Cas9 loss-of-function screening dataset revealed that HDAC2 and HDAC3 exhibited strong genetic dependency in DMG [32, 33]. Therefore, we propose that MS-275, a class I HDACi that is well tolerated and crosses the blood‒brain barrier [44], is a more suitable treatment for DMG. As expected, MS-275 demonstrated potent antitumor effects in preclinical models of DMG. Consistent with other reports, functional studies revealed that MS-275 enhanced cell apoptosis and promoted cell cycle arrest [44–46]. Intriguingly, the CCK-8 assay revealed that primary DMG cells exhibit varying sensitivities to MS-275. Additional studies are needed to explore the underlying mechanisms involved. In addition to its antitumor effect, HDAC inhibition also has a surprising effect on the tumor immune microenvironment. The broad immunopotentiating effects of HDACis include suppressing the infiltration of regulatory T cells, tumor-associated macrophages (TAMs), and myeloid-derived suppressor cells (MDSCs); remodeling the tumor inflammatory landscape; and promoting tumor antigen cross-presentation [47–50]. Numerous studies have shown that HDAC inhibition can alter the tumor cell phenotype, increase the expression of NK-activating ligands, and enhance NK cell-mediated cytotoxicity. In this study, we demonstrated that MS-275, like other HDACis, enhances NK cell cytotoxicity by increasing the expression of NK-activating ligands, such as MICA/B and ULBPs, on the tumor cell surface [17–25]. In addition to altering NK ligand expression in tumor cells, previous study has demonstrated that MS-275 increases the expression of the NK cell surface-activating receptor NKG2D [51]. Additionally, unlike previous studies, we found that MS-275 increased the expression of the NK inhibitory ligand HLA-E. Blocking the HLA-E-NKG2A pathway with monalizumab further increased tumor cell susceptibility to NK cell-mediated killing in vitro. Furthermore, our in vivo assay revealed that, compared with the other treatments, combined treatment with NK-92MI, MS-275, and monalizumab resulted in the most significant tumor growth inhibition effect and the greatest survival benefits. However, in these in vivo experiments, we primarily focused on the therapeutic effects of MS-275 in combination with NK cells and NKG2A blockade, without evaluating the NK cell status by collecting tumor samples through flow cytometry or immunohistochemistry several days after administering NK cells. We propose that effectively monitoring of the presence and activation status of NK cells requires additional mouse models to provide tumor samples at multiple time points post-treatment. In our future research, we plan to conduct more in-depth observations on the presence and activation status of NK cells.

Additionally, we also explored the mechanism by which MS-275 induces elevated HLA-E expression in tumors. As inhibitors of epigenetic regulators, HDACis can promote histone acetylation, regulate chromatin accessibility, and increase gene expression. In this study, we found that the H3K27ac level in the promoter regions of cell proliferation- and apoptosis-related genes changed upon treatment with MS-275, which was associated with its antitumor effect. As expected, the H3K27ac signal was enriched at the HLA-E gene promoter. Gene expression requires transcription factors in addition to chromatin opening. By correlation analysis of gene expression, we speculated that STAT3 is a potential transcription factor of HLA-E. Our results showed that the knockdown of STAT3 in primary DMG cells reduced HLA-E expression. The results of ChIP‒qPCR confirmed that STAT3 functions as a transcription factor regulating HLA-E expression and that HDAC inhibition facilitates this process. However, in contrast to our results, a previous study demonstrated that STAT1α homodimers but not STAT3 bind to GAS-like elements and induce HLA-E transcription [52]. We hypothesized that the differences in the STAT3 antibodies may have led to the different results of the experiments. Furthermore, consistent with previous studies, our results showed that HDAC inhibition promotes the phosphorylation of STAT3 at Y705 and S727. Previous studies indicate that HDAC inhibition promotes LIFR transcriptional activation, which in turn activates the JAK1-STAT3 pathway and the antiapoptotic cascade, a mechanism contributing to tumor resistance to HDAC inhibition [53]. The phosphorylation of STAT3 is required for its dimerization and nuclear translocation, which activates downstream gene transcription. However, inhibiting STAT3 phosphorylation with Stattic did not reduce HLA-E expression in DMG cells, indicating that unphosphorylated STAT3 directly regulates HLA-E expression. Previous studies have shown that STAT3 acetylation at K685 enhances the transcriptional activity of unphosphorylated STAT3 [40]. Given that HDAC1/2/3 can deacetylate STAT3 and that acetylation of STAT3 at K685 enhances DNA binding, transcriptional activity, and nuclear localization [54], we speculated that MS-275 induces STAT3 acetylation, thereby facilitating HLA-E expression. Our results revealed that the HDAC 1/2/3 inhibitor MS-275 significantly increased STAT3 acetylation at K685 and elevated HLA-E expression. Additionally, MS-275-mediated acetylation of STAT3 restored HLA-E expression following STAT3 knockdown. Collectively, our experiments demonstrated that MS-275-induced STAT3 acetylation, rather than phosphorylation, promotes HLA-E expression. However, our study demonstrated that MS-275 specifically affects the acetylation of STAT3 at the K685 site; further rigorous experiments are needed to confirm whether the acetylation of other sites also influences HLA-E expression, considering a limitation of this study.

This study has translational value, as it provides a mechanism-based rationale for combination therapy with the HDACi MS-275. Despite the potent antitumor effects of HDAC inhibition, monotherapy eventually leads to drug resistance [13], highlighting the need for new combination therapy strategies. Given the broad immunopotentiating effects of HDAC inhibition, combining HDACis with immunotherapy for DMG treatment is a promising strategy. Unlike T-cell activation, which depends on antigen processing and presentation, NK cell activation is governed by receptor interactions with target cells and does not cause graft-versus-host disease (GVHD) [55, 56]. Importantly, DMG is more sensitive to NK cells than to T cells [5]. Our research demonstrates that MS-275 treatment enhances NK cells mediated cytotoxicity against DMG cells. Specifically, MS-275 treatment increases HLA-E expression in DMG cells through STAT3 acetylation, thereby improving the efficacy of NK cell-based immunotherapy targeting the HLA-E-NKG2A axis.

## Conclusions

Our findings indicate that MS-275 enhances NK cell-mediated cytotoxicity and sensitizes tumors to monalizumab immunotherapy, suggesting a potential combination strategy for DMG patients.

## Supplementary Information

Supplementary Material 1.

Supplementary Material 2.

## Data Availability

The datasets used and analyzed during the current study are available in the paper’s supplemental material, or from the corresponding author upon reasonable request.
