# Supplementary material for: Targeting the HLA-E–NKG2A axis in combination with MS-275 enhances NK cell-based immunotherapy against DMG
Source: J Exp Clin Cancer Res. 2025 Apr 29;44:133. doi: 10.1186/s13046-025-03390-y (PMC12039099; doi:10.1186/s13046-025-03390-y)
Supplement: Supplementary file 2 — Supplementary Material 2. [file 13046_2025_3390_MOESM2_ESM.docx]

**
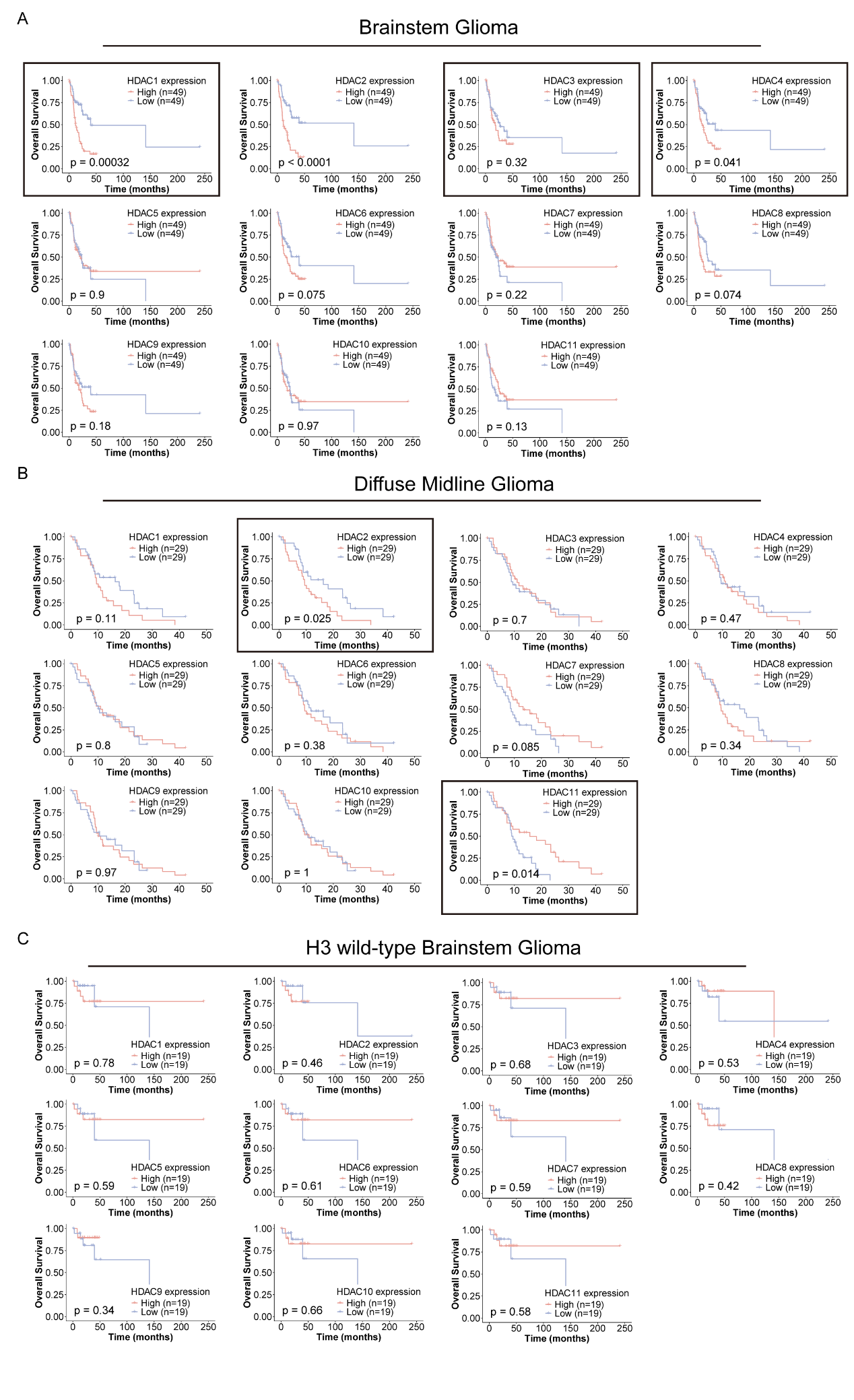
 Figure S1. HDAC expression correlates with survival outcomes in brainstem glioma and H3-Mutant diffuse midline glioma patients, related to Figure 1.**

(A-C) Kaplan-Meier survival curves showing the overall survival of brainstem glioma (BSG) patients (A), H3-mutant diffuse midline glioma patients (B), and H3 wild-type brainstem glioma patients (C). Patients were divided into two groups on the basis of HDAC expression levels, with median expression values serving as cutoffs. The Kaplan‒Meier survival curves were generated using the R packages survival and survminer. P values were calculated via the log-rank test. The black borders indicate statistically significant differences (p<0.05).


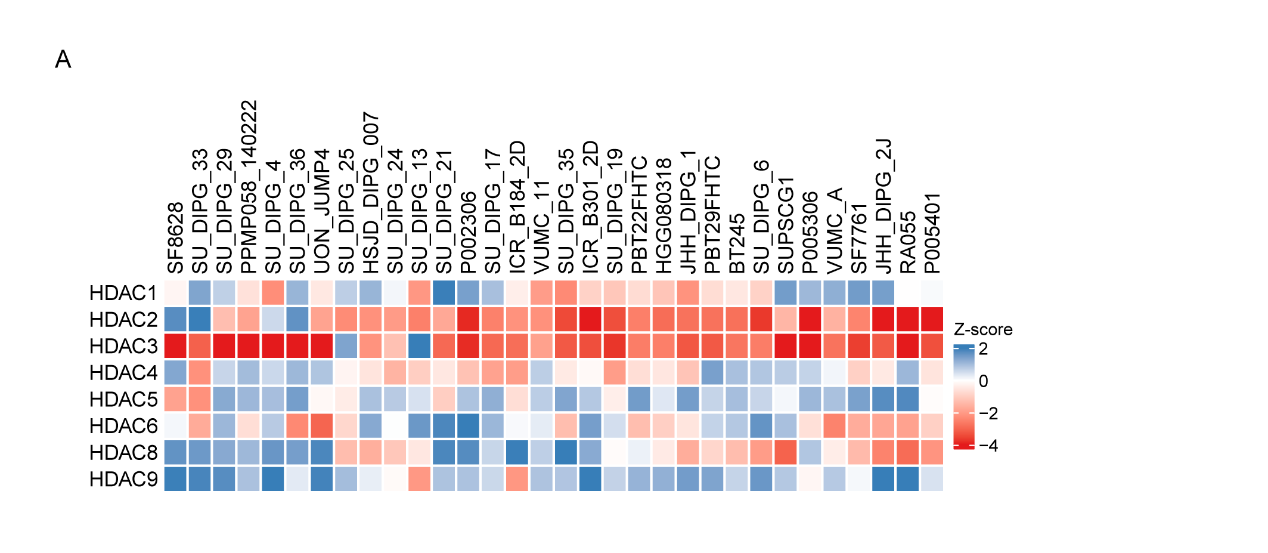


**Figure S2. CRISPR/Cas9 screening in DMG cells, related to Figure 1.**

(A) CRISPR/Cas9 loss-of-function screening across H3K27-altered diffuse midline gliomas (DMGs, n=32).


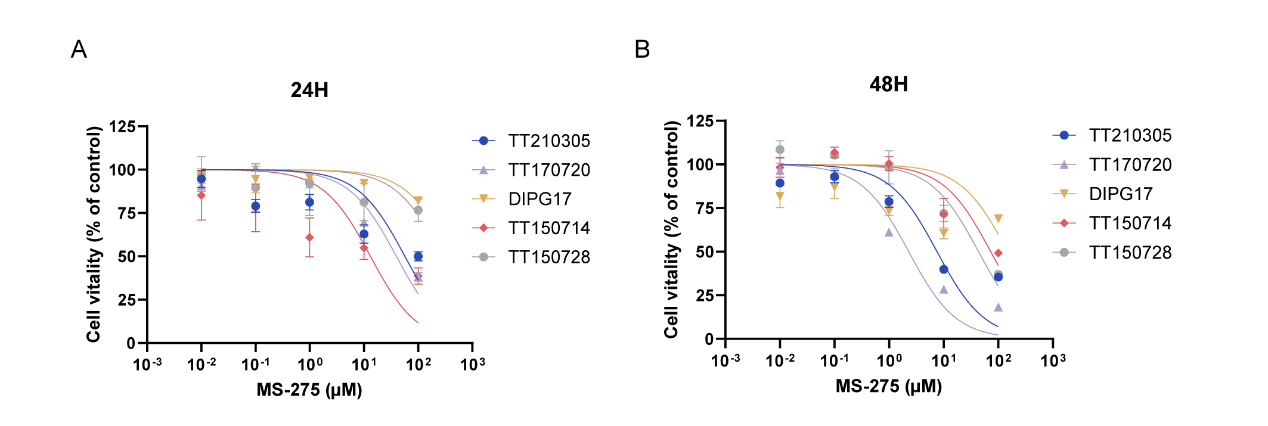


**Figure S3. Evaluation of MS-275 cytotoxicity in other DMG cells, related to Figure 1.**

(A-B) The inhibitory effects of MS-275 on DMG cells (TT150714, TT150728, TT210305, DIPG17, and TT170720) were demonstrated via CCK-8 assays. The cells were treated with various concentrations of MS-275 (0.01–100 µM) for 24 and 48 hours.


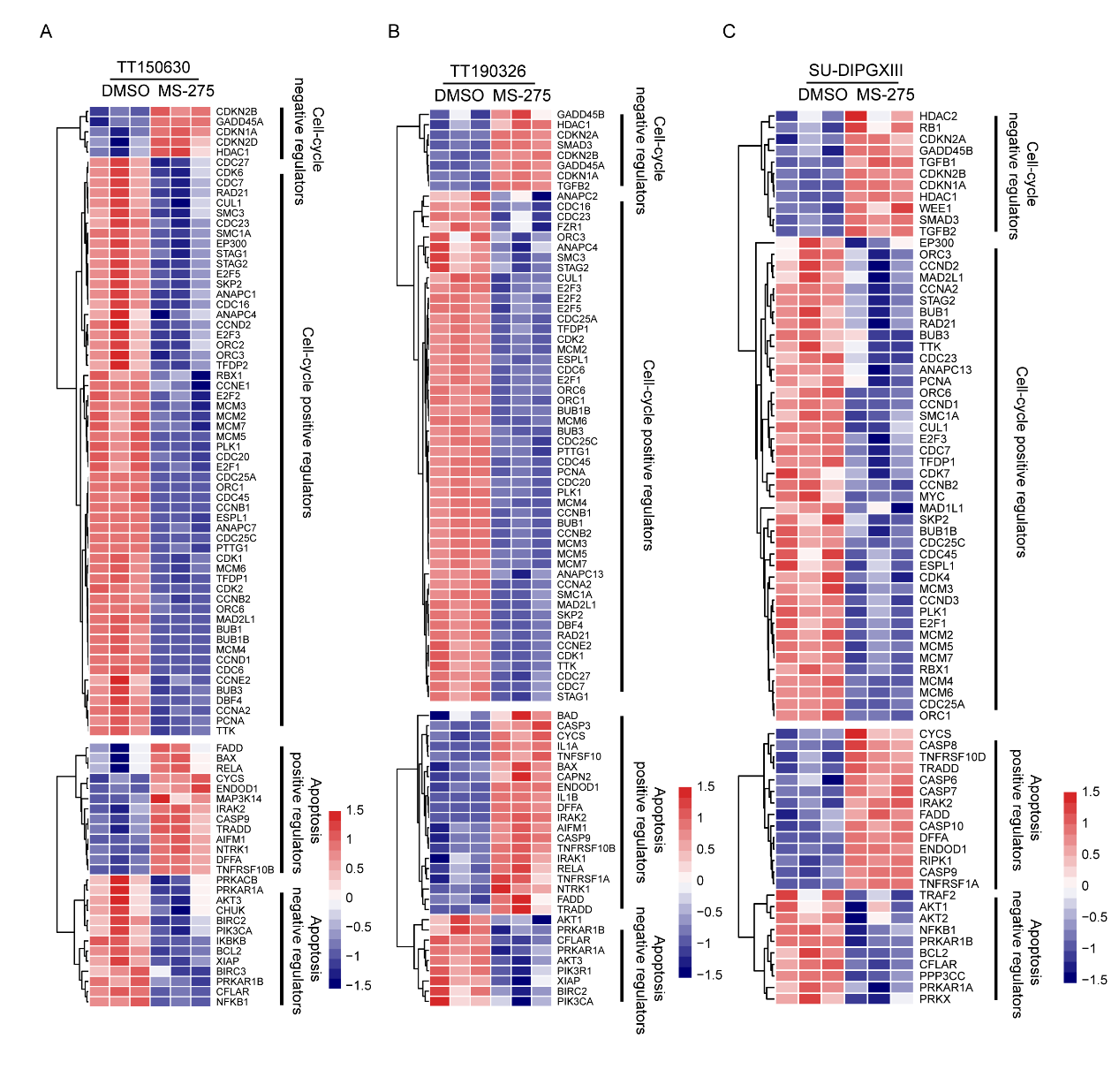
 **Figure S4. MS-275 induces differential expression of cell cycle- and apoptosis-related genes in DMG cells.**

(A-B) Heatmap of cell cycle-related and apoptosis-related genes from RNA-seq transcriptome analysis of TT150630 and TT190326 cells treated with 1 µM MS-275 for 2 days (n= 3 independent experiments).

(C) Heatmap of cell cycle-related and apoptosis-related genes from the GSE110572 dataset of SU-DIPG XIII cells treated with MS-275.


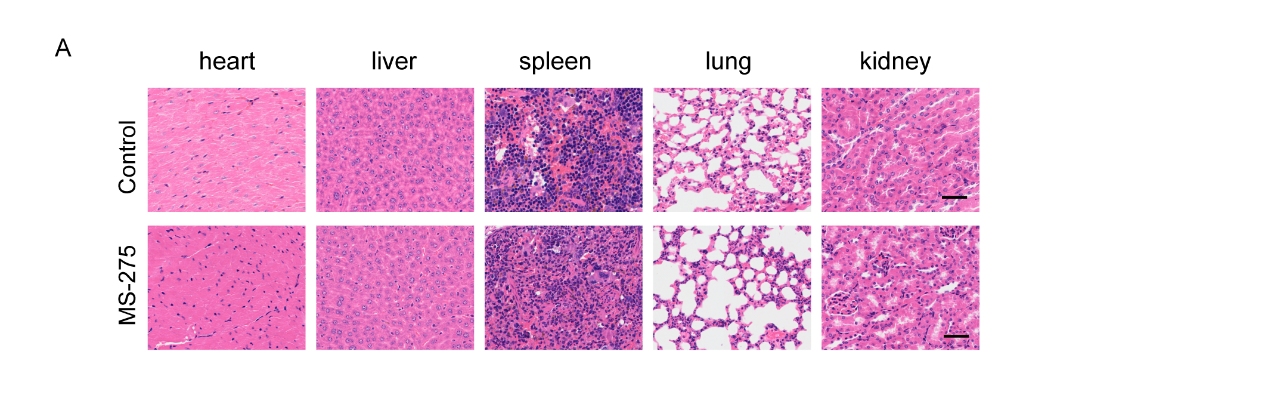


**Figure S5. H&E-staining of the organs of mice xenografted with TT150630-luciferase cells, related to Figure 1.**

(A) H&E-stained sections of the organs of mice xenografted with TT150630-luciferase cells and treated with or without MS-275 in Figure 1. Scale bars, 50 μm.


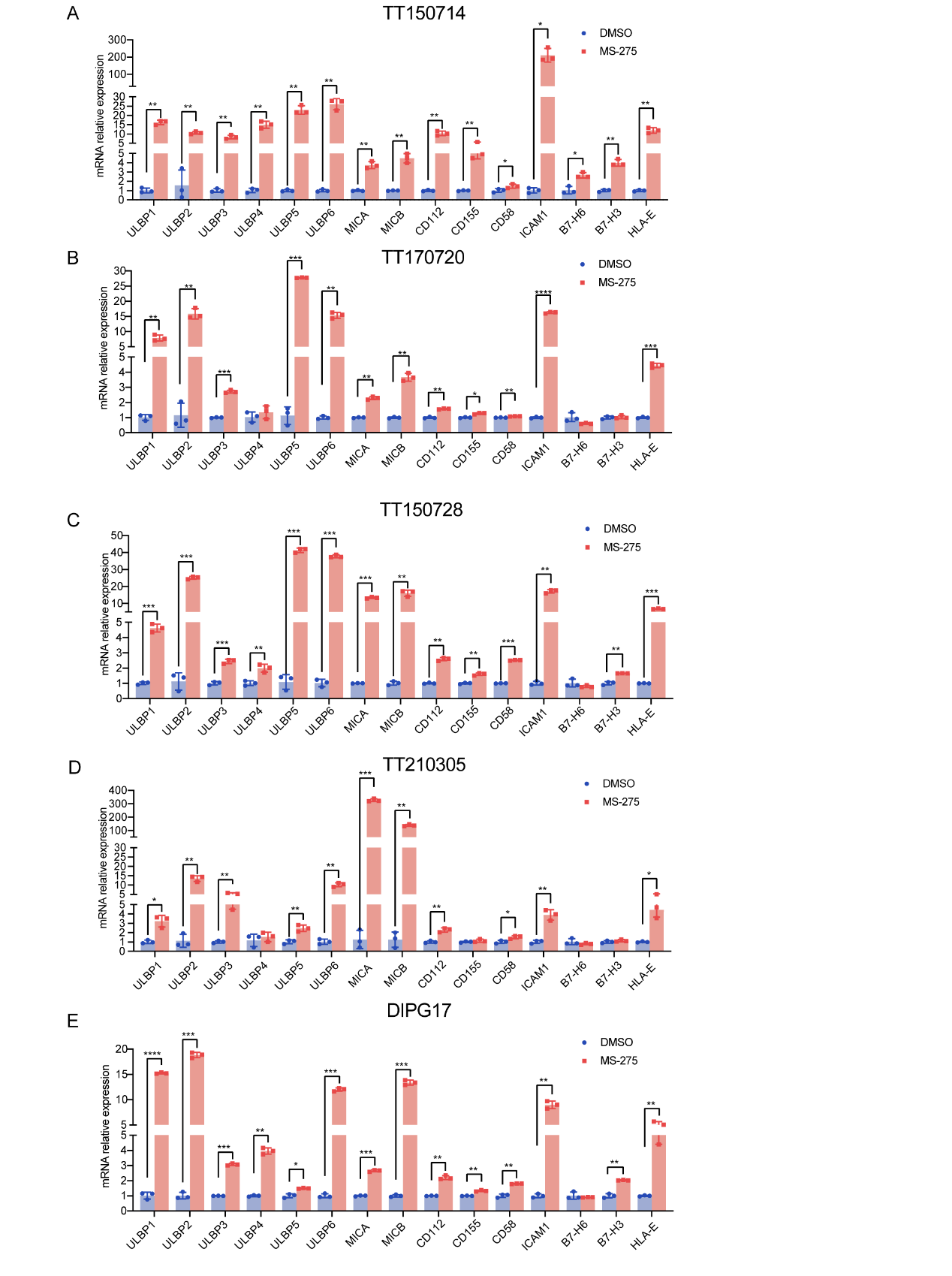


**Figure S6. MS-275 modulates NK cell ligand expression in different DMG cell lines.**

(A-E) RT‒qPCR analysis of the relative expression of NK cell ligands in the indicated DMG cells treated with 1 µM MS-275 for 2 days. Statistical significance was assessed via Student’s t test, with *p < 0.05, **p < 0.01, ***p < 0.001, and ****p < 0.0001.


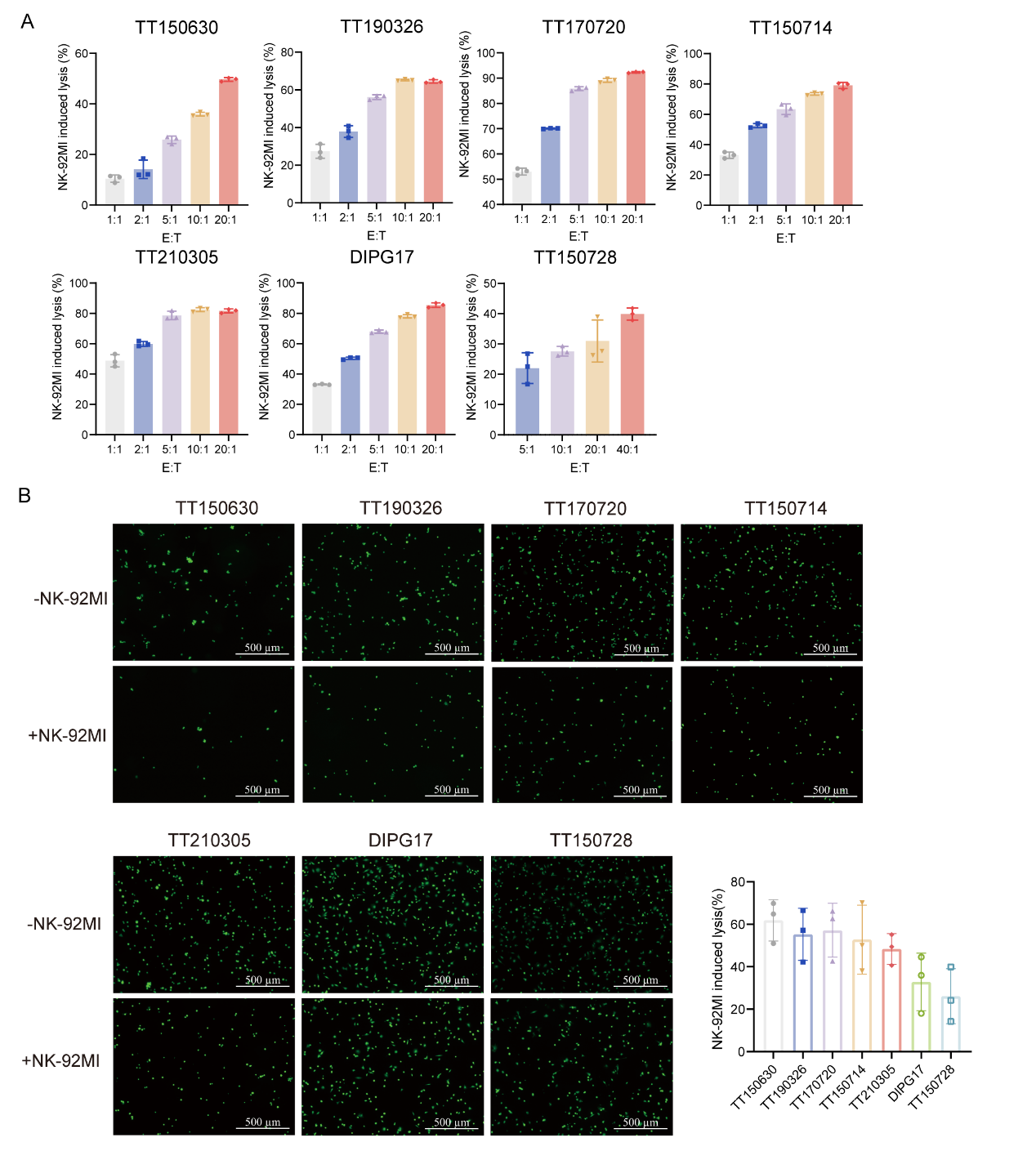


**Figure S7.** **Cytotoxicity of NK-92MI cells against DMG cell lines**

(A) Luciferase-engineered DMG cell lines were cocultured with NK-92MI cells in a 96-well plate at the indicated E:T ratios. After 4 hours of incubation, bioluminescence was measured, and NK cell-induced cytotoxicity (%) was calculated and plotted.

(B) DMG cells were stained with calcein AM and cocultured with NK-92MI cells in 96-well plates at a 1:1 ratio (TT150630, TT190326, TT150714, TT210305, DIPG17 and TT170720) or a 5:1 ratio (TT150728). After incubation for 4 h, fluorescence images were captured using an inverted microscope. Images of DMG cell lines showing a reduction in fluorescence (indicating cell death) are presented. Calcein AM-stained DMG cells without NK-92MI cells served as controls. The quantified results are presented in the plot (right). Scale bars, 500 μm.


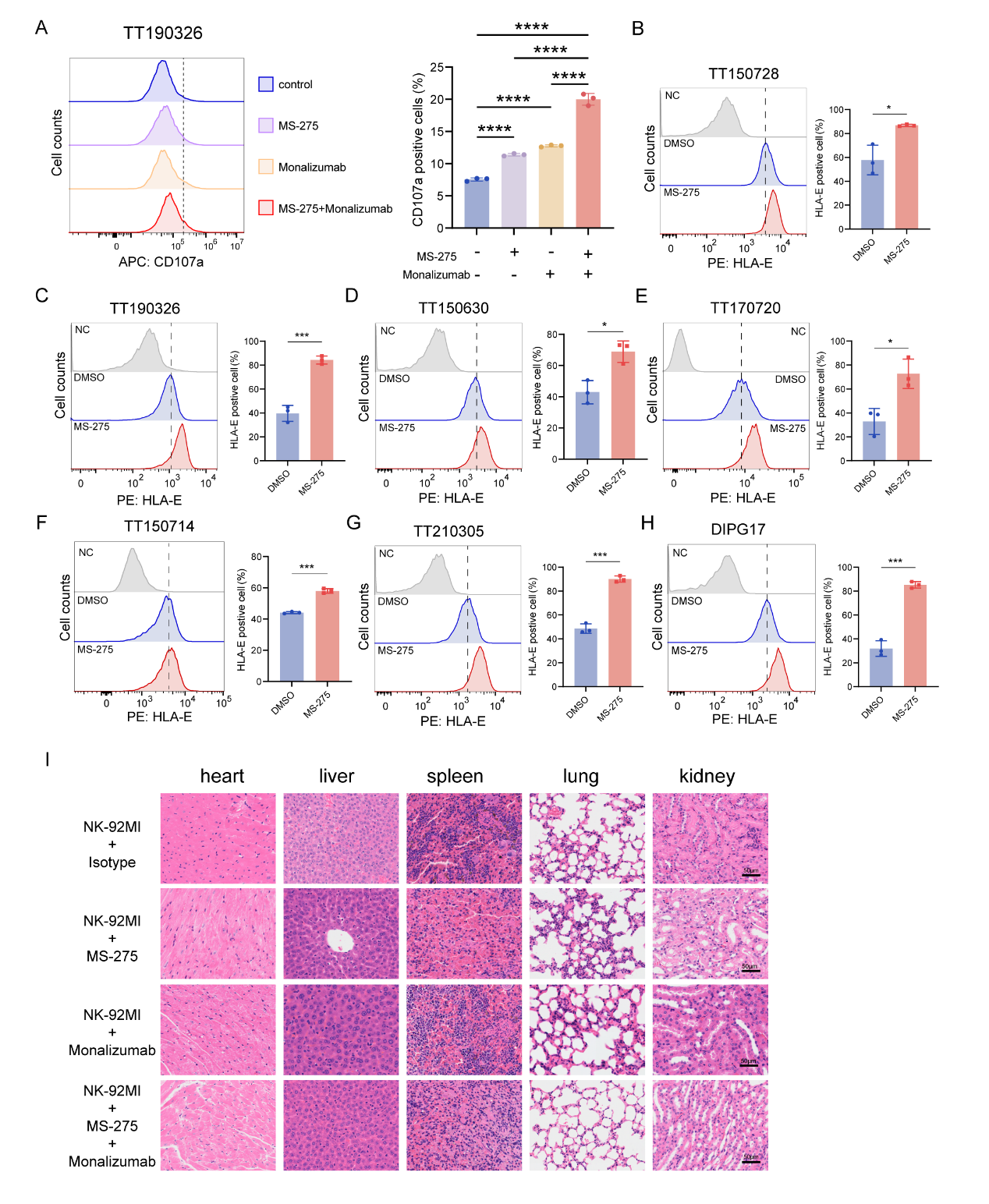


**Figure S8. MS-275 treatment modulates HLA-E expression in DMG cells and histological analysis of xenografted mouse organs**

(A) TT190326 cells were treated with MS-275 (1 µM) for 2 days and then cocultured with NK-92MI cells at a 2:1 ratio for 2 hours. Prior to coculture, NK-92MI cells were preincubated with or without monalizumab (50 μg/ml) for 1 hour at 37°C. After incubation, CD107a expression on the surface of NK-92MI cells was analyzed by flow cytometry to evaluate NK cell degranulation (left). The quantified results are presented in the plot (right).

(B-H) HLA-E expression in the indicated DMG cells treated with 1 μM MS-275 or vehicle for 2 days was measured by flow cytometry (left). The quantified results are shown in the plot (right). Cells that were only stained with isotype control antibodies were used as a negative control (NC).

(I) H&E-stained sections of mouse organs xenografted with TT150630-luciferase cells from each group are shown in Figure 5B. Scale bars, 50 μm.


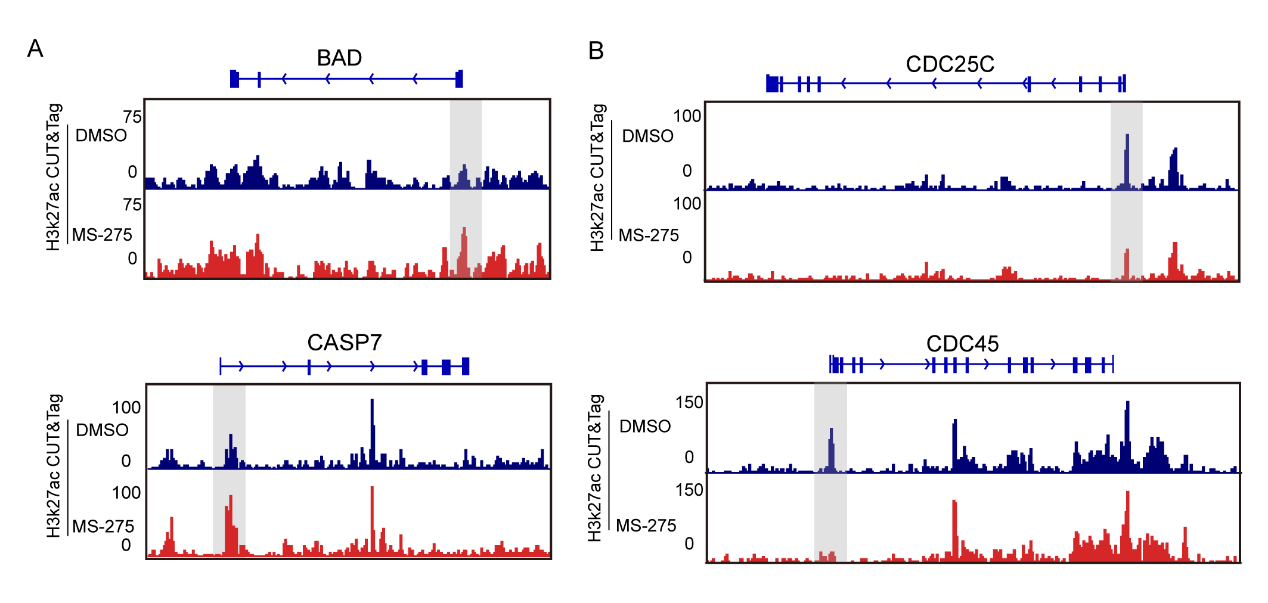


**Figure S9. Alterations in H3K27ac signal in the promoter regions of indicated genes following MS-275 treatment.**

(A-B). Integrated Genomics Viewer (IGV) screenshot showing results of H3K27ac CUT&Tag-seq in peaks at the genomic regions of indicated genes.


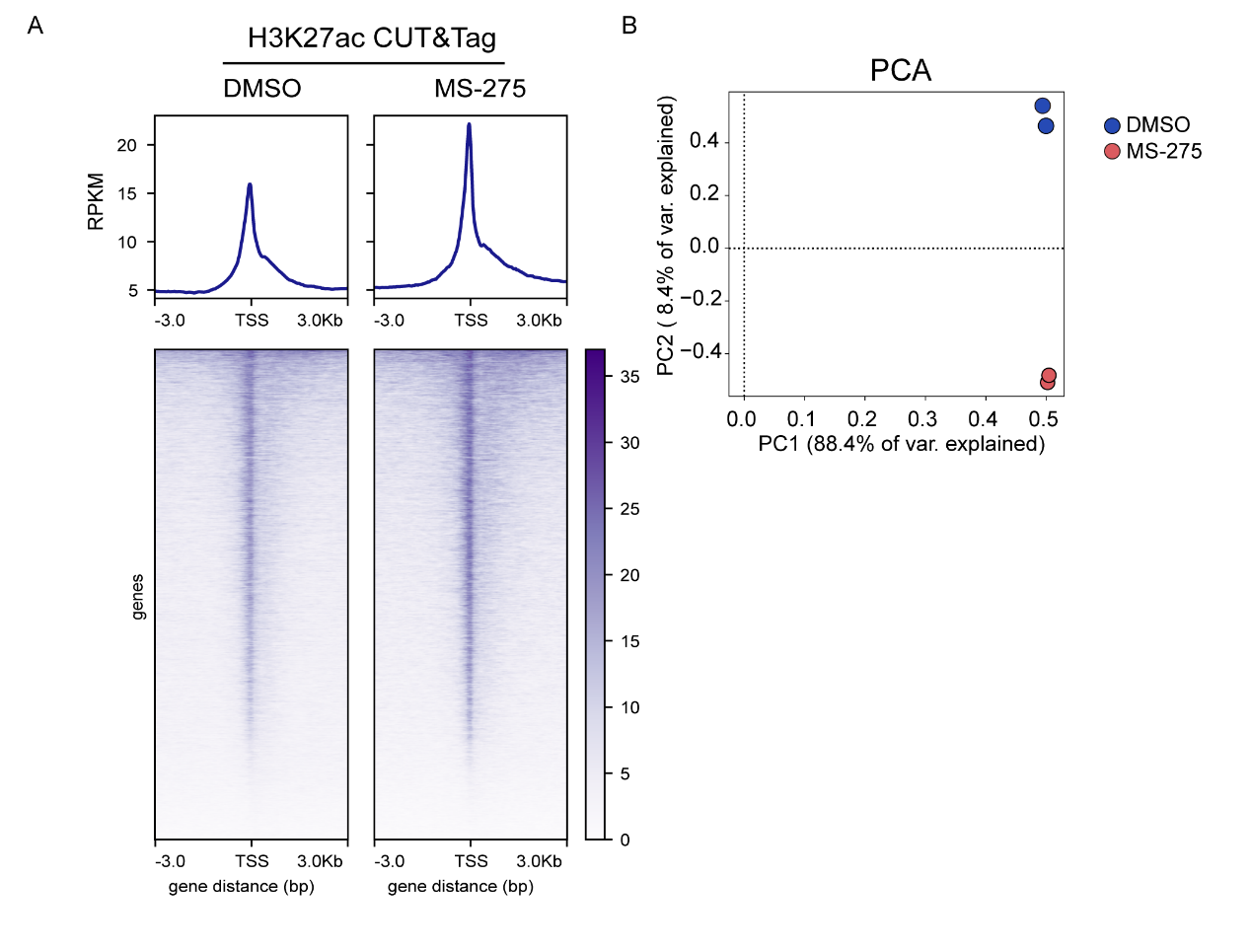


**Figure S10. MS-275 modulates DMG cells epigenetic profile, related to Figure 6.**

(A) Heatmap showing CUT&Tag-seq coverage at sites with altered H3K27ac modifications in MS-275-treated TT150630 cells compared with the DMSO-treated cells.

(B) PCA of CUT&Tag-seq data from DMSO- and MS-275-treated TT150630 cells.

**
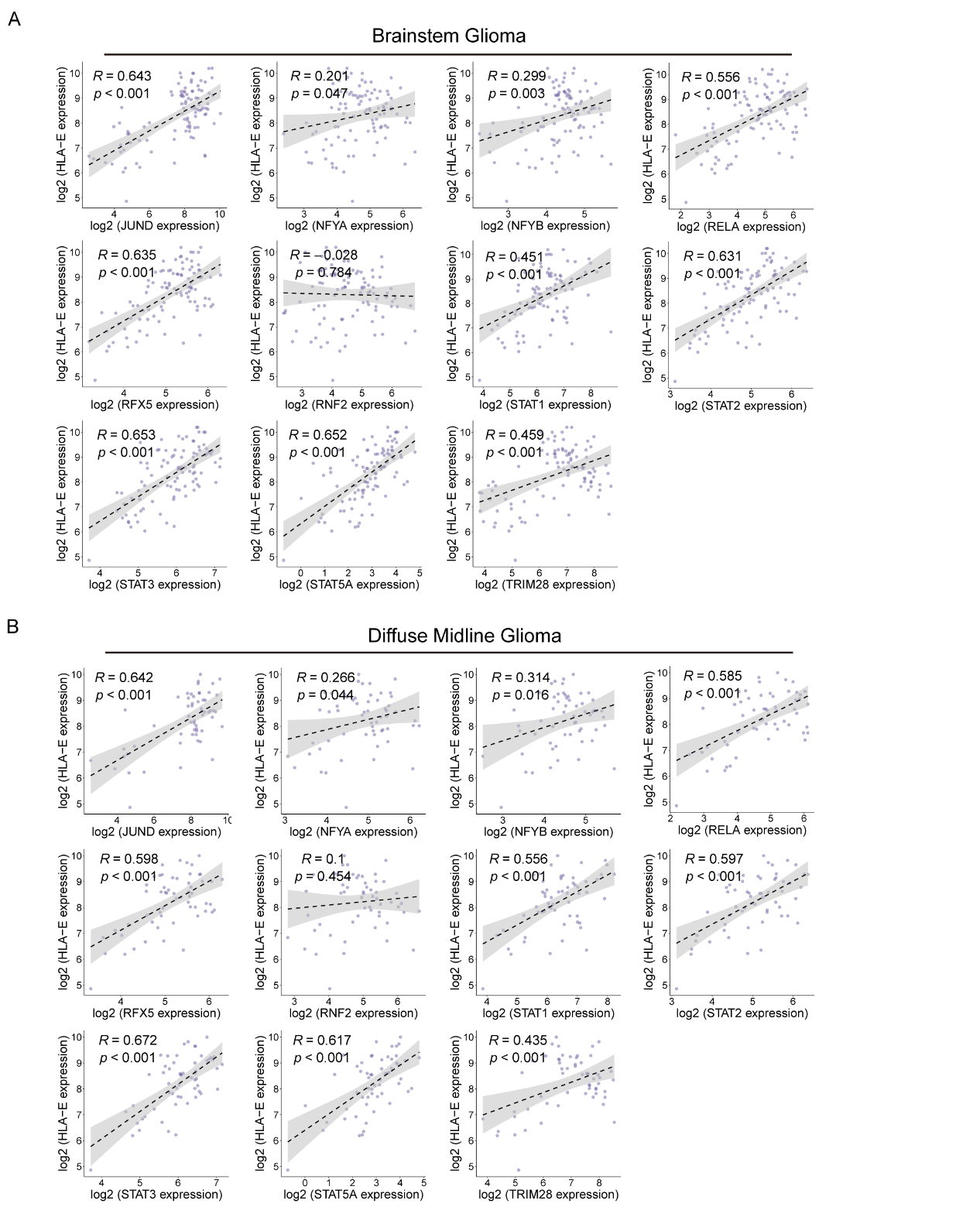
Figure S11. Correlation between transcription factor expression and HLA-E levels in BSG and DMG patients, related to Figure 7.**

(A-B) Pearson’s correlations between 11 potential transcription factors (JUND, NFYA, NFYB, RELA, RFX5, RNF2, STAT1, STAT2, STAT3, STAT5A, and TRIM28) and HLA-E expression in BSG (A) or DMG (B) patients were analyzed. Each data point represents the value of an individual patient.


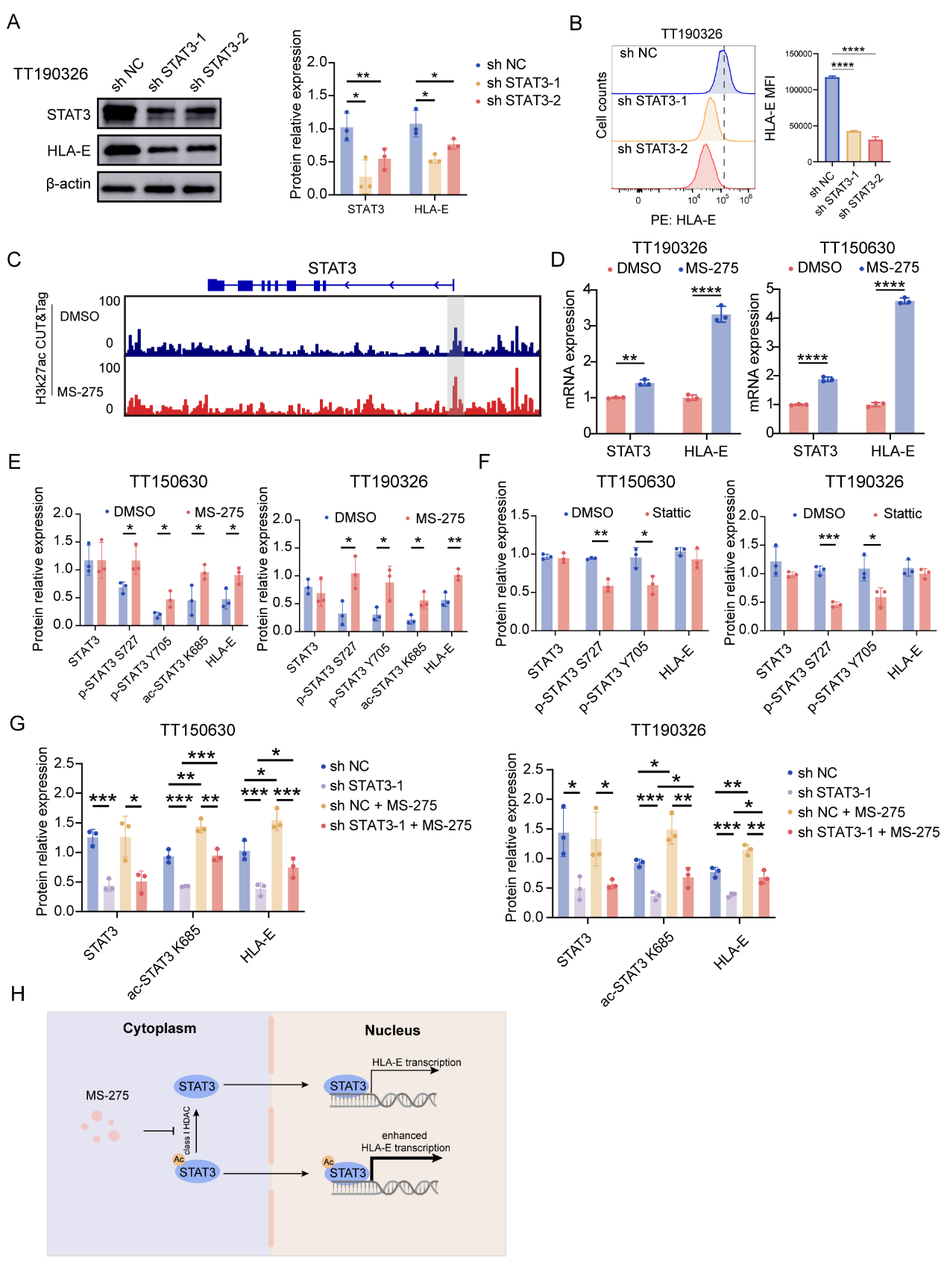
**Figure S12. MS-275 enhances HLA-E expression through acetylation of STAT3, related to Figure 7.**

(A) The protein expression of HLA-E and total STAT3 in TT190326 cells expressing sh-NC or sh-STAT3 was detected via Western blotting. β-Actin was used as the internal control. The quantified results are presented in the plot (right). Statistical significance was assessed via one-way ANOVA with *p < 0.05 and **p < 0.01.

(B) HLA-E expression on the cell surface of TT190326 cells expressing sh-NC or sh-STAT3 was detected via flow cytometry. The quantification of the results is shown on the right. Statistical significance was assessed via one-way ANOVA with ****p < 0.0001.

(C) Integrated Genomics Viewer (IGV) screenshot showing the results of H3K27ac CUT&Tag-seq in peaks at the genomic regions of STAT3.

(D) The mRNA levels of HLA-E and STAT3 in TT190326 and TT150630 cells treated with 1 μM MS-275 for 2 days. Statistical significance was assessed via Student’s t test, with **p < 0.01 and ****p < 0.0001.

(E) The quantified results of the relative protein expression in 7I. Statistical significance was assessed via Student’s t test, with *p < 0.05 and **p < 0.01.

(F) The quantified results of the relative protein expression in 7J. Statistical significance was assessed via Student’s t test, with *p < 0.05, **p < 0.01 and ***p < 0.001.

(G) The quantified results of the relative protein expression in 7K. Statistical significance was assessed via one-way ANOVA with *p < 0.05, **p < 0.01 and ***p < 0.001.

(H) Schematic diagram of the mechanism by which MS-275 enhances HLA-E expression through STAT3 acetylation.
